# Opportunities and Challenges of Using Artificial Intelligence in Predicting Clinical Outcomes and Length of Stay in Neonatal Intensive Care Units: Systematic Review

**DOI:** 10.2196/63175

**Published:** 2025-10-03

**Authors:** Samantha Tudor, Risha Bhatia, Michael Liem, Tafheem Ahmad Wani, James Boyd, Urooj Raza Khan

**Affiliations:** 1 La Trobe University Melbourne Australia; 2 Monash University Melbourne Australia

**Keywords:** artificial intelligence, deep learning, machine learning, neonatal intensive care unit, NICU, predictive modeling, length of stay, clinical outcomes, systematic review

## Abstract

**Background:**

The use of artificial intelligence (AI) in health care has been steadily increasing for over 2 decades. Integrating AI into neonatal intensive care units (NICUs) has promise as it has the potential to reshape neonatal care and improve outcomes. However, challenges such as data quality, clinical interpretation, and ethical considerations may hinder AI’s practical implementation in NICUs.

**Objective:**

This study aims (1) to analyze the current AI research landscape for predicting clinical outcomes and length of stay in the NICU and (2) to explore the benefits and challenges of using AI in the NICU for these predictions.

**Methods:**

A systematic review was conducted across 6 databases—PubMed, Embase, CINAHL, Cochrane Library, Informit, and La Trobe Library—to identify English-language peer-reviewed articles published between January 2017 and March 2023 that focused on the use of AI for predicting length of stay and clinical outcomes for NICU patients. Eligibility criteria excluded studies outside the NICU context or lacking predictive focus. Both prospective and retrospective designs were included. A thematic analysis of AI applications in NICUs from the articles identified was conducted.

**Results:**

A total of 24 studies were included in the review, comprising 15 retrospective and 9 prospective designs. These studies primarily originated from the United States (13 studies), with others from Austria, Taiwan, and other countries. The studies evaluated AI applications in NICU settings to predict comorbidities (18/24), mortality (4/24), and length of stay (2/24). Sixteen studies were in the exploration stage, lacking cohesive AI strategies, while 8 demonstrated systematic exploration but no fully integrated solutions. The synthesis of results identified key applications of AI in NICU care, including data-driven insights and predictive models, advancements in medical imaging, improved risk stratification, and personalized neonatal care. AI showed promise in enhancing diagnostic accuracy and care planning, but significant challenges persist, such as data quality, model generalization, and ethical concerns. No studies reported a fully integrated AI ecosystem, highlighting the need for further research to bridge gaps and realize AI’s transformative potential in neonatal care.

**Conclusions:**

This review highlights the potential of AI in improving NICU care, particularly through predictive models, medical imaging, and personalized interventions. However, the evidence is limited by significant methodological variability, small sample sizes, risk of bias, and a lack of external validation in included studies. Many studies remain in exploratory phases without cohesive AI strategies or integration into clinical practice, limiting the practical applicability of findings. These results underscore the importance of addressing challenges such as data quality, model generalization, and ethical considerations to fully realize AI’s potential in neonatal care. Future research should focus on robust validation, comprehensive implementation strategies, and ethical frameworks to ensure AI's effective and responsible integration into NICU settings.

## Introduction

### Background

Neonatal intensive care units (NICUs) are specialized hospital units providing intensive medical care for critically ill newborns, particularly those born prematurely or with medical and surgical conditions, where close monitoring and specialized interventions and treatment are needed. One in 10 babies is born prematurely or sick, emphasizing the essential role of neonatal care in promoting their well-being and survival [1]. With an estimated 15 million premature births annually and around 1 million child deaths due to preterm complications each year [2], the significance of specialized care is evident. Surviving infants may encounter lifelong challenges, highlighting the need for effective neonatal care to ensure healthy development and improved long-term outcomes.

Within the complex ecosystem of the NICU, health care providers rely on the expertise of skilled clinicians and essential medical devices to deliver specialized care. The implementation of electronic medical records has made vast volumes of data more accessible in recent years, contributing to significant advances in medical research and technology, leading to improved survival rates for NICU patients [3]. However, these advancements come with increased demands on health care resources, including specialized staff, equipment, and facilities. Innovative approaches are essential to address these challenges and enhance overall health care efficacy.

One avenue for improvement is the prediction of the length of stay in the NICU. Accurate length of stay predictions are crucial for resource allocation, discharge planning, and optimizing care pathways [4]. Prolonged stays can have significant implications on neonatal development, parent-newborn interactions, and family well-being [5,6]. Additionally, predicting clinical outcomes is equally important, as it allows for monitoring essential developmental milestones, potential delays or improvements, and the lasting effects of medical interventions on NICU patients [7-12].

Health care providers face several challenges when predicting length of stay or clinical outcomes in the NICU setting. The vast amounts of data, including patient vitals, laboratory results, imaging data, and clinical notes, can be overwhelming to process manually [13]. Limited availability of human resources in high-stress NICU settings further compounds the challenge, where teams must balance heavy workloads and rapid decision-making [14].

Furthermore, the dynamic nature of neonatal care, marked by rapid changes in health status, adds another layer of complexity to accurate predictions. In this context, artificial intelligence (AI) technology emerges as a potential solution. AI refers to the capability of computer systems to carry out tasks that typically require human intelligence, including prediction, learning, and decision-making [15]. While traditional software and other computer systems rely on predetermined rules and instructions to accomplish specific tasks, AI sets itself apart by its capacity to learn from data and continually enhance performance without explicit programming. This capability is primarily achieved through a branch of AI called machine learning [16].

Machine learning encompasses a diverse array of techniques, including supervised learning, where models are trained on labeled datasets (eg, historical patient outcomes); unsupervised learning, which aims to discover hidden structures and patterns within unlabeled data; and reinforcement learning, a paradigm focused on optimizing decision-making through iterative interactions with an environment and the receipt of rewards. Deep learning, a specialized form of machine learning, uses multilayered neural networks that mimic the human brain’s processing abilities to uncover complex relationships in large datasets. In the context of this study, these techniques are especially valuable for predicting neonatal outcomes, such as length of stay or health conditions, using data from electronic medical records. By leveraging machine learning and deep learning, AI has the potential to transform decision-making in NICUs, providing more accurate, timely, and personalized insights to support neonatal care [12].

In health care, predictive analytics powered by AI has proven effective for disease diagnosis and risk stratification, offering insights from patient data, medical records, and imaging results [17,18]. However, AI integration in the NICU environment is not without its challenges. Concerns related to data privacy, algorithm bias, and the need for transparent and interpretable models raise ethical considerations [19,20]. Understanding these challenges is essential for the responsible and successful implementation of AI for predictive analytics in neonatal care.

Despite the growing body of research, existing reviews in this field [12,21,22] have primarily focused on technical performance metrics, such as model readiness and diagnostic accuracy, while overlooking critical themes such as stakeholder engagement, ethical considerations, and data integration. For example, Schouten et al [22] focus on model evaluation but overlook broader integration opportunities, Adegboro et al [21] highlight health impacts but neglect barriers like trust and collaboration, and McAdams et al [12] emphasize predictive performance without addressing implementation challenges such as data quality and explainability. To address these gaps, this study synthesizes recent research (2017-2023) through a thematic lens, providing actionable insights into opportunities such as predictive modeling and personalized neonatal care, as well as challenges such as ethical issues and stakeholder trust. A systematic review design was chosen to ensure a comprehensive and methodologically rigorous synthesis of available literature on AI applications in NICU settings. This approach allows for structured evidence appraisal and thematic synthesis across diverse study designs and outcomes, providing robust and evidence-based recommendations to guide clinical, policy, and research decisions in this rapidly evolving field.

The importance of advancing research in this area becomes even more apparent when considering that other researchers have highlighted the lack of investigation into the practical implementation of AI in intensive care units. For instance, Vellido et al [23] lament the paucity of research on success factors for implementing machine learning in intensive care units, while Kim et al [24] point out that although challenges to implementing these technologies are acknowledged, they remain underexplored. This study seeks to address these gaps by providing a comprehensive and updated synthesis to guide future research and practical implementation in NICUs.

### Research Focus and Aims

This review aimed to address the research question, “What are the opportunities and challenges in using AI in the NICU to predict length of stay and clinical outcomes?”

The objective was to analyze the current AI research landscape for predicting clinical outcomes and length of stay in the NICU, exploring the benefits and challenges of using AI in the NICU for these predictions. This will help identify gaps in AI applications for predicting clinical outcomes and length of stay in the NICU and offer recommendations for future research.

## Methods

### Methodology Overview

A systematic review methodology was applied to provide a robust and transparent synthesis of evidence related to AI applications in neonatal intensive care. This approach followed the PRISMA (Preferred Reporting Items for Systematic Reviews and Meta-Analyses) guidelines and involved systematic search, screening, data extraction, quality appraisal, and thematic analysis across multiple databases [25]. The review was tailored to the digital health context by focusing on studies with predictive outcomes in the NICU, aligning with current research priorities and ensuring methodological depth.

To enhance relevance and address recent advances, a targeted supplementary search of literature published post–March 2023 was conducted to identify key developments for discussion. These findings were integrated thematically into the Discussion section.

### Database Search

English language peer-reviewed studies between January 2017 and March 2023 were searched in 6 databases (Embase, MEDLINE, CINAHL, Cochrane, Informit, and La Trobe Library). The search used relevant search strings and Medical Subject Headings (MeSH). The keywords included artificial intelligence, NICU, length of stay, and outcome, as detailed in Table 1.

**Table 1:** Search method and number of results per database.

Database	Search method	Results, n
Ovid (MEDLINE and Embase)	(exp Artificial Intelligence/ OR AI OR “artificial intelligence” OR “machine learn*” OR “deep learn*” OR “neural network*”) AND (exp Intensive Care, Neonatal/ OR exp Infant, Premature/ OR exp Infant, Premature, Diseases/ OR exp Intensive Care Units, Neonatal/) AND ((“length of stay” OR “LoS”).mp. OR (“outcome” OR “prognosis”).mp.)	249 (64 and 185)
CINAHL	((artificial intelligence OR ai OR a.i. OR “machine learning” OR “deep learning”) AND (nicu OR “neonatal intensive care unit” OR “special care” OR “baby unit” OR “newborn intensive care”)) AND ((“length of stay” OR los OR “inpatient stay” OR “time in hospital” OR “time to discharge”) OR (outcomes OR prognosis)) AND (English Language) AND (Published Date: 20170101-20230131)	21
Cochrane	(MH “Artificial Intelligence”) AND (MH “Intensive Care Units, Neonatal”)	1
Informit	“Artificial Intelligence” AND “Intensive Care Units, Neonatal”	0
La Trobe Library	((Al OR “artificial intelligence” OR “machine learn*” OR “deep learn*” OR “neural network*”) AND (NICU OR “neonatal intensive care unit*” OR “newborn intensive care unit*”) AND (“length of stay” OR “LoS” OR “outcome” OR “prognosis”)) AND (English language and last 5 years)	541

### Eligibility Criteria

The inclusion criteria focused on NICU patients and the adoption of AI technology for predicting outcomes and length of stay. Outcomes were defined as measures related to the adoption of AI technology for predicting length of stay and clinical outcomes in NICU patients, including mortality, morbidity, growth and development, and other clinical parameters up to 5 years of age. This framework was consistently applied during the eligibility assessment to ensure a clear and systematic approach. Both retrospective and prospective studies were included for a comprehensive view. Retrospective studies offer historical insights through electronic health records, while prospective studies provide real-time data for dynamic observations. The combination ensured a comprehensive evaluation, considering AI's opportunities and challenges in the NICU. Studies within the last 5 years, in English, and with full-text availability were included, aiming to understand AI's role in predicting NICU outcomes and length of stay. Multimedia Appendix 1 details the reasons for exclusion of studies during the screening process.

### Quality Appraisal

The quality appraisal process was conducted using a structured approach to ensure rigor and minimize bias. Multiple authors were involved in the selection and coding of studies to enhance reliability and reduce subjective influence. Initially, ST screened titles and abstracts for relevance, whereas URK and RB independently reviewed the screening, following which discrepancies were resolved through discussion or consultation during regular meetings.

The included studies underwent quality assessment using modified WHO (World Health Organization) guidelines [26] and preferred QCC (Quality Checklist for Clinical Case Series) [27] due to varied study designs. Multimedia Appendix 2 [28-51] contains detailed ratings, evaluating bias and quality.

### Data Extraction

Relevant data from the selected studies were systematically extracted and recorded in Microsoft Excel. A customized template, designed collaboratively by the research team specifically for this systematic review, was used for data extraction. The template included key features, such as study characteristics (background, methods, results, conclusions, and impact), study details (type of study, keywords, country, and number of participants), technology-related information (technology type and algorithm or model details), outcome (length of stay or outcome), evaluation measures, as well as insights on potential opportunities, challenges, discussed gaps, further research needs, and suggested improvement areas. The extracted data were organized and analyzed for synthesizing key findings and identifying and coding emerging themes relating to the research objectives using Thomas and Harden’s method of thematic analysis [52]. Studies identified for full-text review were coded to identify recurring concepts, which were grouped into descriptive themes. Analytical themes, such as those related to challenges and opportunities, were developed to interpret the findings and provide actionable insights. This method ensured a structured and transparent synthesis of diverse qualitative data. The identification and development of themes were conducted iteratively, with refinements made after each round of discussions among the research team to achieve consensus and ensure accuracy. This collaborative process ensured a comprehensive and balanced synthesis of the evidence, leveraging diverse expertise to strengthen the review's findings.

### Assessment of Maturity Levels in AI Adoption

The maturity levels of AI adoption in the included studies were assessed using a structured framework defining “exploring,” “emerging/activating,” or “integrated ecosystem” stages [53]. These stages were defined as follows:

**Explorer (exploring):** This stage reflects ad hoc efforts to leverage AI in health care, with no established benchmarks or strategic frameworks. Stakeholders, including policymakers and governments, may have begun exploring AI's potential but have not yet developed or drafted relevant policies or guidelines.

**Emergent (emerging or activating):** At this stage, efforts to use AI in health care are more systematic and linked to a national AI strategy with clearly defined priorities. Policies and guidelines supporting AI inclusion in public health are drafted, demonstrating progress toward structured integration.

**Leader (integrated ecosystem):** This stage represents the highest maturity level, where AI adoption is embedded in national strategies and aligned with public health goals. Policies and guidelines are implemented and continuously updated, reflecting a well-established and operational AI ecosystem in health care.

These indicators encompassed several dimensions, such as the presence of national AI strategies, systematic exploration of AI applications, and the existence of policies or guidelines supporting AI integration. Careful consideration was given to the content, including mentions of AI strategies, the level of systematic exploration, and any references to comprehensive policies or frameworks. Each article was then categorized according to the maturity level that most accurately reflected its alignment with these dimensions. ST initially performed the categorization, which was then reviewed by URK and ML.

This methodical process ensured that the categorization of articles was based on discernible criteria, promoting consistency and reliability in assessing the state of AI adoption in neonatal care research. By applying these definitions and criteria, the assessment provided a clear understanding of the progress and gaps in AI maturity across the included studies.

### Cohort Size Classification

Participant cohort sizes were categorized as small (fewer than 100 participants), medium (100 to 1000 participants), and large (more than 1000 participants) to standardize the classification of studies based on the scale of their population data.

### Performance Measure Evaluation Categories

In evaluating the performance measures for the AI models used in the included studies, a systematic approach was used to rate their effectiveness based on the results obtained and the type of measure used, supported by statistical norms for machine learning [54]. The evaluation results were categorized as follows.

#### Area Under the Receiver Operating Characteristic Curve, Correlation, and Sensitivity and Specificity

**Excellent (0.9-1):** AI models within this category exhibited exceptional predictive accuracy and discrimination capabilities. They demonstrated a high degree of confidence in distinguishing between outcomes.

**Good (0.8-0.9):** Models in this range displayed strong predictive performance. They possessed a notable ability to differentiate between outcomes, indicating reliability in predictions.

**Moderate (0.7-0.8):** AI models within this range demonstrated moderate predictive performance. While reasonably discriminative, there was room for enhancement in their accuracy.

#### Normalized Average Root Mean Square Error

**Excellent (0-lower values are better):** AI models achieving lower values for normalized average root mean square error (RMSE) demonstrated superior predictive accuracy. Lower RMSE values indicated predictions that closely aligned with actual outcomes.

This systematic rating approach allows comprehensive evaluation of the performance of AI models in predicting clinical outcomes and length of stay in the NICU. It provided a structured framework for assessing the reliability and accuracy of predictions based on the specific evaluation measures used in this study.

### Reporting

The PRISMA 2020 checklist was used to guide the reporting of this systematic review, ensuring a comprehensive and transparent presentation of the methodology and findings (Multimedia Appendix 3). Certain items (such as statistical synthesis or meta-analysis) were not applicable due to the heterogeneity of study designs and outcomes, but all relevant PRISMA elements were addressed in line with best practices for qualitative systematic reviews.

## Results

### Study Selection

**Figure 1:**
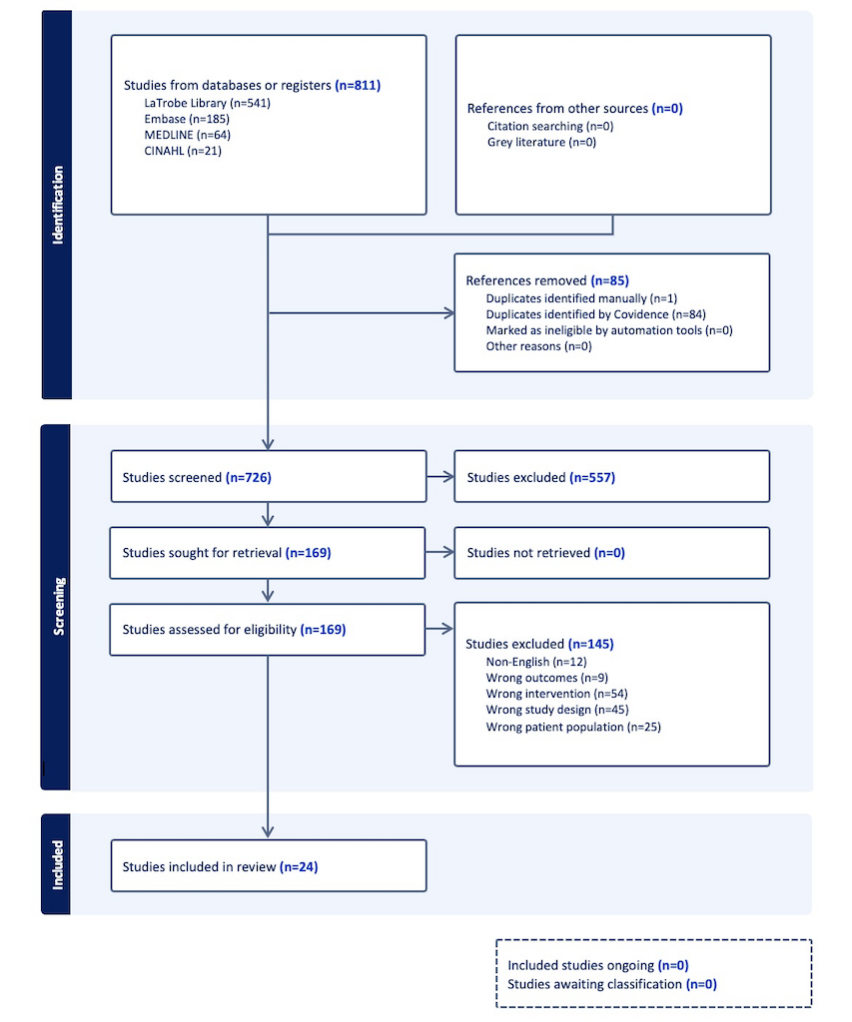
PRISMA (Preferred Reporting Items for Systematic Reviews and Meta-Analyses) flow diagram.

A total of 811 studies were obtained and imported into the Covidence Systematic Review Software (Veritas Health Innovation). Among them, 85 duplicates were removed, resulting in 726 studies for screening. Out of the initial 726 studies, 169 were retained following a title and abstract review. The final selection comprised 24 articles deemed appropriate and included following a full review. A PRISMA flow diagram (Figure 1) illustrates the process of article selection and exclusion.

### Study Characteristics

#### Key Features of Included Studies

**Figure 2:**
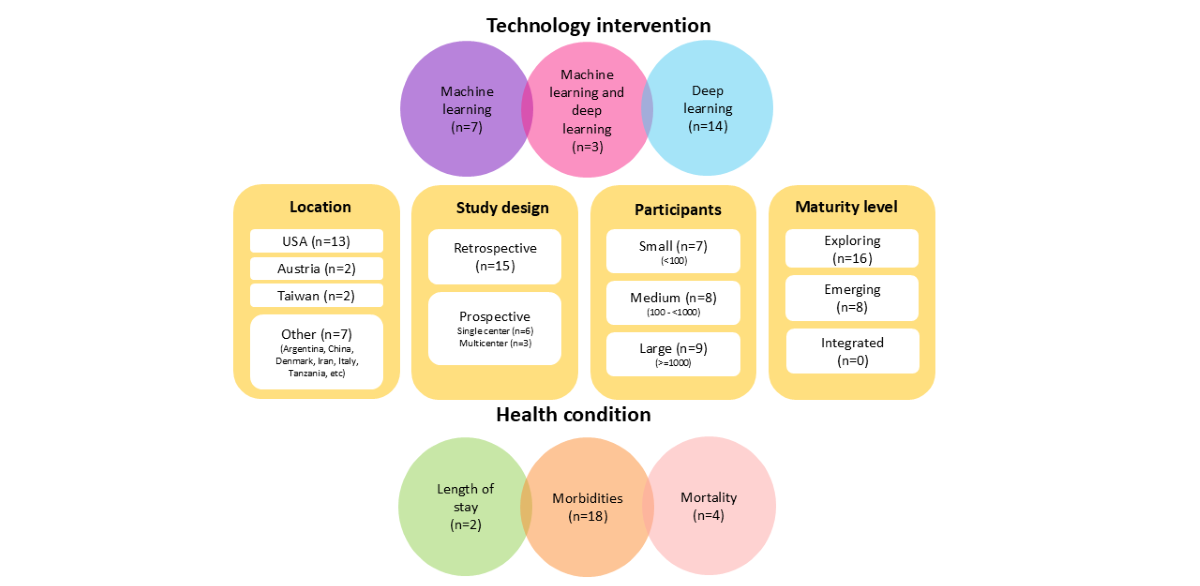
Study characteristics of AI predictions for NICU patients (n=24), including technology interventions, location, type of study design, number of participants, the AI maturity level, and the outcome predicted. AI: artificial intelligence; NICU: neonatal intensive care unit.

Multimedia Appendix 4 [28-51] provides detailed characteristics of the included studies in a tabular format. The 24 included studies, as seen in Figure 2, include various technology interventions in NICU research: 7 using machine learning [28-34], 3 combining machine and deep learning [35-37], and 14 exclusively focusing on deep learning [38-51]. Geographically, 13 studies are from the United States [28,29,33,37,38,40,42,43,45,47-50], with others from Austria (2) [30,41], Taiwan (2) [44,46], and 6 in different countries including Argentina [32], China [36], Denmark [34], Iran [51], Italy [35], and Tanzania [31], along with 1 multinational dataset [39]. Study designs include 15 retrospective, 6 prospective single-center, and 3 multicenter studies. Participant cohorts varied, with 7 studies featuring small cohorts (fewer than 100), 8 with medium (100 to 1000), and 9 with large cohorts (1000+).

Of the 24 included studies, 16 fall under the “exploring” category, lacking a cohesive AI strategy [28,29,31,32,36,38-40,42,44-47,49-51]. Eight articles were classified as “emerging/activating,” showing systematic exploration but without a fully established AI ecosystem [30,33-35,37,41,43,48]. None were categorized under “integrated ecosystem,” indicating that a mature and fully established AI ecosystem in neonatal care remains a future aspiration.

The included 24 studies primarily analyze 3 key outcomes: length of stay (2 instances), morbidities (18 studies), and mortality (4 studies). These characteristics offer insights into the diverse range of studies in AI's current NICU research.

#### Temporal Technological Trends

**Figure 3:**
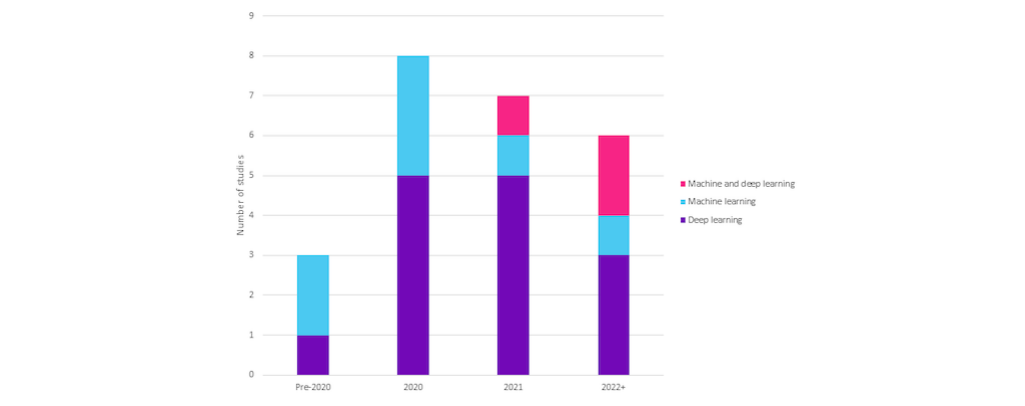
Temporal technology trends of included studies (totaling 24) across different technologies—machine learning, deep learning, and a combination of both—based on their publication years.

The 24 included studies depict temporal technology trends (Figure 3), revealing shifts in preference within neonatal care research. In 2020 and 2021, five studies emphasized deep learning [39-41,43-45,47,49-51], signifying its growing recognition in predicting outcomes. This trend continues in 2022, with 3 studies opting for deep learning [38,46,48]. Post-2021, studies integrate machine learning and deep learning [35-37] methods, reflecting evolving research methodologies and the importance of advanced AI, particularly deep learning, in neonatal care predictions.

#### Morbidities Studied

**Figure 4:**
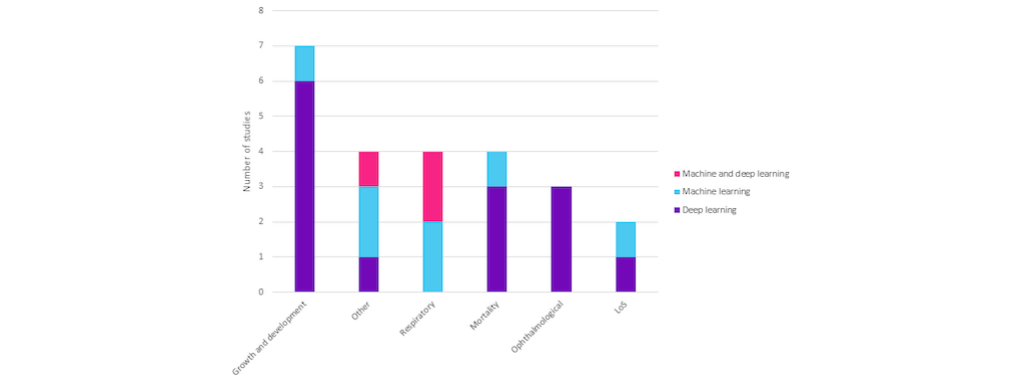
Outcome type by technology type, illustrating the distribution of 24 included studies across different outcome types (growth and development, other, respiratory, mortality, ophthalmological, and LoS) and technology types (machine learning, both machine learning and deep learning, and deep learning). The chart provides insights into the focus areas of these studies and the technology used to address specific neonatal care outcomes. LoS: length of stay.

The 24 studies in this review outline AI technology's applications in predicting specific NICU clinical outcomes (Figure 4), revealing prevalent trends. “Growth and development” leads with 7 studies, mostly using deep learning [30,38,41-43,49,50], emphasizing AI's role in assessing infant development. “Ophthalmological” outcomes are studied in 3 exclusively deep learning-driven articles [39,40,44]. “Respiratory” outcomes involve 4 studies, equally leveraging deep learning and machine learning studies [32,34-36], underscoring their relevance in neonatal respiratory health. The “other” category spans a variety of outcomes, with different AI techniques used. “Mortality” is studied by 4 articles, mostly using deep learning [31,45,48,51]. Lastly, “length of stay” involves 2 studies, evenly split between deep learning and machine learning [28,46]. Overall, deep learning emerges as the predominant approach across categories, showcasing its diverse applicability in neonatal care. This comprehensive analysis accentuates AI's complex role in enhancing infant health and well-being. Multimedia Appendix 5 [28-51,55-66] summarizes clinical outcomes categories and descriptions of relevant studies.

#### Predicting Outcomes Evaluation Measures

**Figure 5:**
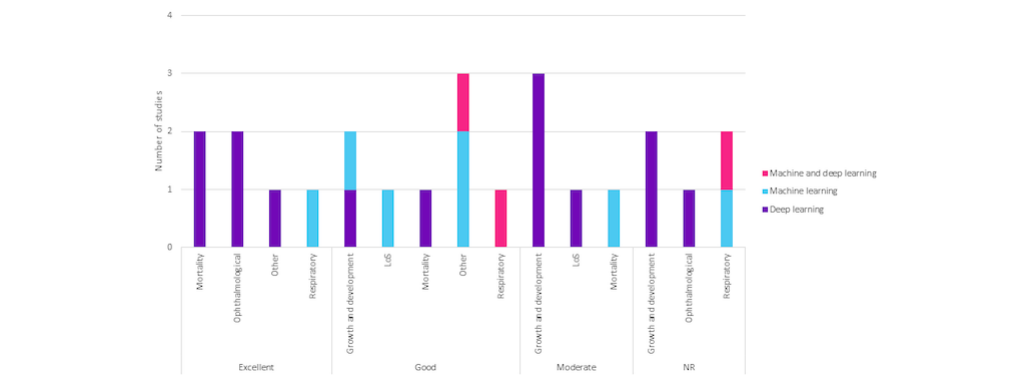
Performance outcome by type of AI, displaying the performance assessment of AI models (machine learning, deep learning, and a combination of both) across different neonatal care outcome types (growth and development, other, respiratory, mortality, ophthalmological, and LoS). Performance is categorized into 4 levels: excellent, good, moderate, and NR. The chart provides insights into the quality of AI models in predicting specific neonatal care outcomes across different AI types and outcome categories. LoS: length of stay; NR: not reported.

The 24 studies use diverse predictive model evaluation measures (Table 2) to assess machine or deep learning model accuracy in predicting outcomes or classifications in neonatal care. Figure 5 shows AI model performance across outcome categories, labeled “excellent,” “good,” “moderate,” or “not reported.” In the “excellent” category, deep learning excelled in “other,” “ophthalmological,” and “mortality” outcomes, while machine learning performed well in “respiratory” outcomes. “Good” models, primarily deep learning, demonstrated strength in “growth and development” and “mortality,” while machine learning and deep learning showed promise in “other” and “respiratory” outcomes. “Moderate” performance was observed in deep learning for “growth and development” and “length of stay,” and in machine learning for “mortality.” Notably, 5 studies [32,35,40,41,49] did not report performance, including 2 that specified the measure used (eg, AUROC [area under the receiver operating characteristic curve] and error percentiles) but did not provide the results [35,41], and 3 that did not report either the measure or the results [32,40,49]. This figure highlights AI strengths in various outcomes, indicating research directions and areas needing improvement.

**Table 2:** Evaluation measures.

Evaluation measure	Number of studies	Study
Area under the receiver operating characteristic curve	15	[28,29,35-39,42,43,45-48,50,51]
Correlation (*r*)	1	[30]
Error percentiles	1	[41]
Normalized average root mean square error	1	[44]
Sensitivity and specificity	3	[31,33,34]
Not reported	3	[32,40,49]

Table 2 demonstrates various evaluation measures for predicting neonatal outcomes. Among these, the most common metric is AUROC, used in 15 studies. Other measures such as correlation (*r*), error percentiles, and normalized average RMSE were less frequent, each found in a single study. Sensitivity and specificity were the focus of 3 studies. However, specific evaluation measures were not reported in 3 instances, showcasing diverse approaches to assessing AI models in neonatal care studies.

#### Performance by Outcome and Type of AI

Figure 5 provides an overview of the performance of AI models in predicting neonatal outcomes, categorized as “excellent,” “good,” “moderate,” or “not reported,” and further differentiated by the type of technology used. In the “excellent” category, deep learning models demonstrated exceptional predictive abilities across various outcomes, including “other,” “ophthalmological,” and “mortality,” while machine learning models were effective in predicting “respiratory” outcomes. The “good” category saw deep learning models excelling in “growth and development” and “mortality,” with machine learning and deep learning models performing well in “other” and “respiratory” outcomes. Machine learning models also demonstrated proficiency in predicting “length of stay.” “Moderate” AI models, predominantly deep learning, showed moderate performance in “growth and development” and “length of stay,” while machine learning models displayed moderate performance in 1 “mortality” study.

### Insights Into Potential Opportunities for AI in NICUs

#### Opportunity Themes for AI in NICU Research: Overview

Opportunities for AI applications in NICU have been themed into 5 categories (summarized in Table 3), from enhancing NICU care through data-driven insights and predictive models to using advancements in medical imaging, improving risk stratification, and personalizing neonatal care and interventions.

**Table 3:** Opportunity themes and findings.

Study	Application of AI^a^ in the NICU^b^	Advancements in medical imaging	Data-driven insights and predictive models	Improving understanding and risk stratification	Personalized neonatal care and intervention
Iyer et al (2022) [30]	✓		✓		✓
He et al (2023) [36]	✓				
Ali et al (2022) [38]	✓				
Lin et al (2022) [46]	✓		✓		
Lee et al (2021) [45]	✓		✓		
Chen et al (2021) [39]	✓	✓	✓		
Gschwandtner et al (2020) [41]	✓				
He et al (2020) [43]	✓	✓			
Braun et al (2020) [28]	✓		✓	✓	✓
Choi et al (2020) [40]	✓	✓			
Saha et al (2020) [50]	✓	✓			✓
Huang et al (2020) [44]	✓	✓			
Hamilton et al (2020) [29]	✓		✓	✓	
He et al (2018) [42]	✓			✓	
Kausch et al (2022) [37]	✓				
Verder et al (2021) [34]	✓				
Patel et al (2022) [48]	✓		✓		
Ruixiang et al (2021) [49]	✓		✓		
Shalish et al (2017) [33]	✓		✓		
Sheikhtaheri et al (2021) [51]	✓				
Ofman et al (2019) [32]	✓		✓	✓	
Amodeo et al (2021) [35]	✓		✓	✓	✓
Kovacs et al (2021) [31]	✓				
Lure et al (2021) [47]	✓	✓	✓		✓

^a^AI: artificial intelligence.

^b^NICU: neonatal intensive care unit.

#### Advancements in Medical Imaging

Several studies within the selection highlight medical imaging advancements. Two studies focus on retinopathy of prematurity (ROP), showcasing the role of deep learning in standardizing disease assessment and predicting visual outcomes posttreatment [39,44]. Choi et al [40] also explored deep learning for ROP severity assessment, highlighting broader medical implications. He et al [43] introduced a multitask deep transfer learning model for early neurodevelopmental outcome prediction in preterm infants, emphasizing imaging's predictive role. Saha et al [50] used deep learning convolutional neural networks to forecast motor outcomes via brain diffusion MRI (magnetic resonance imaging) data. Lastly, Lure et al [47] used machine learning to distinguish neonatal conditions, enhancing clinical decision-making. These studies highlight medical imaging's potential in early diagnosis and monitoring of neonatal conditions.

#### Data-Driven Insights and Predictive Models

Data-driven insights and predictive models in neonatal care have the potential to enhance outcomes [28-30,33,39,45,46,48,49]. They contribute to care planning by using machine learning to predict factors like length of stay, disease severity, and mortality risk, aiding health care providers [30,45,46]. Some explore risk assessment, detecting, and mitigating severe neonatal morbidities [28,29,50]. Two introduce adaptive machine learning algorithms for dynamic patient status adaptation [35,48]. Lastly, Ruixiang et al [49] highlight data-driven insights' transformative role in advancing neonatal research and medical care by improving predictions, care planning, and risk assessment.

#### Improving Understanding and Risk Stratification

The theme presents a cohesive effort toward understanding neonatal conditions and individual risk stratification through data-driven approaches. Three articles focus on machine learning–driven risk assessment for conditions like bronchopulmonary dysplasia (BPD), in-hospital length of stay, and mortality risk [42,45,46]. Leveraging extensive datasets, these studies offer critical insights into associated risk factors, enabling early intervention. Additionally, 3 articles delve into NICU usage trends [28], severe morbidity risk [29], and chronic lung disease determinants in very low birth weight infants [32]. Two studies emphasize dynamic machine learning algorithms for mortality prediction and pulmonary hypertension (PH) [35,48]. Together, these studies deepen our understanding of neonatal conditions, reshaping disease paradigms and paving the way for more effective interventions.

#### Personalized Neonatal Care and Intervention

Numerous studies contribute to the theme of “personalized neonatal care and intervention,” extending the horizons of individualized care for neonates. These studies explore personalized care plans and interventions. They investigate trends in NICU usage [28], assess risk factors for severe neonatal morbidity [29], predict motor outcomes [50], and differentiate between critical neonatal conditions [47]. By leveraging predictive models and continuous monitoring, these studies seek to tailor care strategies to the specific needs of each neonate. This data-driven approach holds the potential to revolutionize neonatal care by optimizing interventions and leading to better clinical outcomes.

### Insights Into Potential Challenges for AI in NICUs

#### Challenge Themes Overview

Literature indicated some concerns that span multiple facets of AI application in NICU, ranging from data quality and quantity issues to clinical interpretability, model generalization, clinical and diagnostic variability, and ethical and regulatory considerations. Table 4 provides a condensed overview of the key thematic challenges encountered across the various included studies.

**Table 4:** Challenge themes and findings.

Study	Data quality and quantity challenges	Clinical interpretability and usability	Model generalization and validation	Clinical and diagnostic variability	Ethical and regulatory challenges
Iyer et al (2022) [30]		✓		✓	
He et al (2023) [36]	✓		✓		
Ali et al (2022) [38]	✓				
Lin et al (2022) [46]					
Lee et al (2021) [45]	✓			✓	
Chen et al (2021) [39]		✓	✓		✓
Gschwandtner et al (2020) [41]		✓	✓	✓	
He et al (2020) [43]		✓			
Braun et al (2020) [28]		✓			
Choi et al (2020) [40]	✓		✓	✓	
Saha et al (2020) [50]	✓		✓	✓	
Huang et al (2020) [44]	✓				
Hamilton et al (2020) [29]					
He et al (2018) [42]	✓				
Kausch et al (2022) [37]		✓			
Verder et al (2021) [34]	✓		✓	✓	
Patel et al (2022) [48]					✓
Ruixiang et al (2021) [49]	✓				
Shalish et al (2017) [33]		✓		✓	
Sheikhtaheri et al (2021) [51]	✓		✓	✓	
Ofman et al (2019) [32]			✓	✓	✓
Amodeo et al (2021) [35]			✓	✓	
Kovacs et al (2021) [31]		✓			✓
Lure et al (2021) [47]					

#### Data Quality and Quantity

Several studies face recurring challenges with health care data in the NICU, including issues related to quality, quantity, and availability [34,36,38,40,42,44,45,49-51]. Addressed by multiple articles, these challenges span data quality's influence on predictive models, the need for larger datasets, variability due to data quality, and concerns about both data quality and quantity. The authors highlight these challenges in developing effective machine learning solutions for neonatal care. Overcoming these hurdles is crucial for successful machine learning implementation, ultimately enhancing health care outcomes for neonates.

#### Clinical Interpretability and Usability

Challenges in clinical interpretability and usability arise as machine-generated insights need integration into clinical practice. Studies emphasize the need for standardized measures for neurodevelopmental assessment, ethical data sharing, complex functional connectivity estimation, user-friendly tools in low-resource settings, and distinguishing specific illnesses [28,30,31,33,37,39,41,44]. Overcoming these challenges is crucial for meaningful AI integration in the NICU and its adoption by health care professionals, and improving health care outcomes for these patients.

#### Model Generalization and Validation

The challenge of ensuring AI model reliability across diverse populations in neonatal care is evident. He et al [36] advocate for external validation of AI models for BPD severity prediction across various populations. Chen et al [39] stress the need for adaptable and generalizable models, particularly for diverse patient demographics and different camera systems in ROP. Similarly, Choi et al [40] focus on creating versatile deep learning scales for ROP applicable across various centers. Saha et al [50] emphasize the importance of generalizing predictions for diverse neonatal populations in motor outcome prediction. Verder et al [34] highlight the necessity of generalizing and validating models for accurate predictions in diverse clinical scenarios for BPD. Sheikhtaheri et al [51] discuss the validation of predictive models for neonatal deaths in NICUs, recognizing the need for external validation. Ofman et al [32] note the challenge of studying disease determinants across multiple centers in chronic lung disease. Amodeo et al [35] explore the complexities of predicting PH outcomes in a diverse patient population.

#### Clinical and Diagnostic Variability

Handling clinical and diagnostic variability is a critical challenge in the NICU, with particular significance in fields such as ophthalmology and critical care, where even subtle variations can have a profound impact on outcomes [30,37,40,45,47,50]. Iyer et al [30] highlight the need for standardized, objective, and scalable measures for neurodevelopmental assessment, contrasting with current subjective and nonscalable methods. Similarly, Lee et al [45] note the necessity for a larger training dataset and consideration of site-specific differences to improve model performance. In ophthalmology, Choi et al [40] highlight the diagnostic variability and subjective quantification of severity levels, hindering the interpretation of clinical trial data. Saha et al [50] raise concerns about the limited sample size and heterogeneity of brain injuries, which may lead to overfitting and poor prediction performance. Furthermore, Shalish et al [33] point out practice variability and uncertainty in defining extubation failure, complicating clinical decision-making.

Addressing imbalanced data and the need for standardized physiological testing are highlighted by Ofman et al [32] and Sheikhtaheri et al [51], respectively. Moreover, Amodeo et al [35] underscore the challenges in measuring lung vascularization accurately, given the techniques' reproducibility issues and the impact of hemodynamic changes during neonatal transition. These challenges collectively illustrate the complexity and variability inherent in neonatal care, underscoring the importance of developing robust AI models and data-driven solutions to enhance precision and reliability in clinical decision-making.

#### Ethical and Regulatory Challenges

The integration of machine learning in neonatal care introduces ethical and regulatory complexities. Chen et al [39] emphasize the ethical challenge of data sharing, weighing the practicality against privacy concerns in multi-institutional datasets. The study highlights regulatory hurdles in validating models across diverse populations and camera systems. Patel et al [48] note the regulatory risks of overfitting single-site datasets and ethical concerns around addressing missing data. They also note the need to evaluate model performance with evolving clinical cohorts, which has regulatory implications. Ofman et al [32] underline ethical and regulatory issues linked to nonstandardized physiologic testing in NICUs, impacting research and clinical practices. Kovacs et al [31] note the ethical concern of deploying tools in resource-limited settings, balancing computational limitations with clinicians' interpretability. These articles emphasize the intricate ethical and regulatory challenges in AI integration in neonatal care, requiring careful navigation for ethical standards and regulatory compliance.

## Discussion

### Key Findings

The objective of this systematic review was to analyze the current AI research landscape for predicting clinical outcomes and length of stay in the NICU, exploring the benefits and challenges of using AI in the NICU for these predictions. The surge in studies between 2020 and 2021 reflects a growing recognition of AI's potential to reshape neonatal care, possibly accelerated by the COVID-19 pandemic's impact on health care systems and driving innovative solutions to mitigate disruptions. The sustained research momentum, including studies in 2022, signifies an enduring commitment to exploring AI's role in the NICU.

This study contributes to the growing field of AI in NICUs by addressing gaps left by previous reviews and advancing the discourse on critical factors for successful AI adoption. Unlike earlier reviews, which predominantly focused on technical aspects and model performance, this study emphasizes emerging themes, including the importance of explainability, multidisciplinary collaboration, and stakeholder engagement. For instance, while [22] and [12] underscore technical barriers and model readiness, they lack focus on real-time data integration and actionable pathways for clinical implementation. This study builds on their findings by highlighting the transformative potential of AI in enabling predictive modeling, personalized care, and multidimensional data synthesis, essential for advancing NICU practices. Furthermore, this review synthesizes research through a broader thematic lens and incorporates the most recent evidence from 2017 to 2023. The systematic review design ensures a methodologically rigorous and transparent synthesis of the latest research, enabling relevance and depth in addressing practical challenges such as ethical concerns, stakeholder trust, and system integration. By using structured quality appraisal and thematic analysis across diverse study types, this review offers actionable insights that are not only academically robust but also critically important for guiding health care professionals, policymakers, and technologists in implementing AI solutions tailored to the unique complexities of NICU environments.

In terms of technologies used, deep learning emerges as a dominant technology across various clinical outcomes, highlighting its effectiveness in automatically identifying relevant features from raw data, especially in medical imaging and clinical analysis. However, the field's maturity in AI integration within neonatal care remains in the early exploration stage, lacking a mature and cohesive ecosystem.

The growing presence of various national guidelines and policies highlights the progress of AI implementation within health care [67-69]. However, there is a need to prioritize the development of a national AI strategy tailored for neonatal care due to the unique challenges of this environment. Specific considerations for neonatal care include the need for specialized models that account for the unique physiology and comorbidities of premature infants, as well as the heightened need of ensuring patient safety and data privacy in a patient population that have continuous and complete medical records from birth.

The study reveals substantial opportunities for the use of AI in the NICU to predict length of stay and patient outcomes. These cover diverse areas: advancements in medical imaging, data-driven insights and predictive models, improved understanding and risk stratification, and personalized neonatal care and intervention.

The first opportunity focuses on AI's potential to transform neonatal care in the NICU by leveraging machine learning to perform predictive analytics to help inform the clinician during the clinical decision-making process, aiding in earlier interventions. However, there are research gaps in translating AI's theoretical effectiveness into real-world NICU settings [3-6,70-74]. This brings model generalization and validation, the third challenge theme, which highlights the importance of ensuring AI models perform reliably across diverse patient populations. External validation across different populations is necessary for reliability and adaptability. The unique patient populations and scenarios in the NICU demand tailored approaches for model generalization and validation, vital for trustworthy predictions.

The second opportunity highlights the use of AI in NICU medical imaging. Six studies primarily use deep learning to analyze medical images, notably for conditions such as ROP. AI-driven imaging enhances accuracy, facilitating early intervention and personalized care plans. These findings not only affirm anticipated AI benefits in medical imaging but also underline its relevance in the NICU, particularly focusing on neonatal conditions. AI-powered imaging is key for precise diagnosis, standardized disease assessment, and predicting critical outcomes, aligning closely with literature expectations [15,75,76]. It enables tailored, timely care plans for individual neonatal needs, a significant advantage in this setting. The challenge here is handling clinical and diagnostic variability in these settings, where even subtle variations can have an impact on outcomes. Addressing this challenge involves the development of models and algorithms that can adapt to diverse clinical contexts, accounting for nuances that might otherwise be overlooked. It calls for an ongoing effort to refine models and diagnostic tools to accommodate the inherent variability in neonatal care, ultimately improving the precision and reliability of clinical decision-making.

The third and fourth opportunities highlight the transformative power of data-driven insights and predictive models in the NICU. Thirteen studies within these themes leverage AI to translate health care data into actionable insights, aiding decision-making. They encompass predictive modeling, spanning length of stay, disease severity, and mortality risk. These studies deepen the comprehension of neonatal conditions and their determinants, aligning with the literature's focus on advancing scientific understanding and medical care in the NICU [44,76-81]. By using data-driven approaches, they pave the way for redefining disease paradigms, ultimately enhancing interventions and care strategies. The challenge here is data quality and quantity, as it involves acquiring high-quality, sufficient data for AI model training. Data incompleteness and inconsistency, common issues in health care data, align with existing literature. Overcoming these challenges requires advanced algorithms and robust data management strategies within the NICU [80].

The final opportunity, personalized neonatal care and intervention, signals a significant shift in NICU health care. Studies focusing on tailoring care plans and interventions for individual neonatal needs explore personalized care plans, risk assessment, predicting motor outcomes, and distinguishing critical neonatal conditions. These efforts, using predictive models and continuous monitoring, aim to optimize interventions. Personalized care plans carry substantial implications, potentially reducing unnecessary treatments, cutting health care costs, and improving clinical outcomes. These align with literature stressing the importance of individualized neonatal care [71,82]. This is closely related to challenges of clinical interpretability and usability, as well as managing clinical and diagnostic variability. Clinical interpretability and usability focus on the transparency and practicality of AI-driven models within the NICU. Health care providers in the NICU demand accurate predictions and a clear understanding of AI outputs.

### Recent Advances in AI Research in NICU Settings

Building on the findings of this review, more recent AI research in NICU settings (post-2023) demonstrates continued advancement across key clinical domains, particularly neurological development, ophthalmological conditions, and respiratory outcomes. A growing body of literature emphasizes the integration of longitudinal and multisource datasets to strengthen predictive modeling. For instance, studies focused on neurodevelopmental outcomes have begun incorporating nationwide longitudinal clinical and sociodemographic data to more accurately identify risks of cognitive delay [83,84]. In the respiratory domain, pilot studies have explored the early detection of BPD by analyzing volatile organic compounds in exhaled breath, identifying novel biomarkers to support early diagnosis and intervention [85]. Similarly, machine learning techniques have been applied to uncover predictive clinical variables spanning the perinatal and neonatal periods, enhancing early risk stratification for respiratory morbidity [86]. Recent developments also highlight a shift toward multifactorial models that enable personalized care planning. Notably, the Mendelian Phenotype Search Engine (MPSE) uses natural language processing and machine learning to automatically identify high-risk neonates likely to benefit from whole genome sequencing, improving diagnostic yield and enabling timely interventions within the first 48 hours of NICU admission [87]. Parallel advancements in ophthalmology include AI-assisted diagnosis for ROP, with recent studies validating model outputs through clinician review to enhance diagnostic accuracy and trust [88,89]. These post-2023 developments align with our review findings and reinforce the trajectory toward integrated, real-time, and patient-centered AI solutions in neonatal care.

Regardless of any application, ethical and regulatory considerations and challenges remain in all applications. It emphasizes the complex landscape requiring careful navigation. Key ethical concerns include data sharing dilemmas and safeguarding patient data privacy and security. Regulatory hurdles, especially in validating AI models across diverse populations and settings, require adaptable frameworks. Only 4 studies directly mention these ethical and regulatory challenges, suggesting a potential research gap in the NICU field of AI, requiring more exploration and consideration. Ensuring responsible AI adoption is pivotal for equity in health care access and maintaining high standards of care.

Recent studies confirm these challenges and suggest strategies to address them. For instance, learning models, enabling collaborative training across institutions without sharing raw data, have emerged as a practical solution to mitigate privacy risks while enhancing model robustness [21,90]. Large-scale neonatal research networks, such as the Vermont Oxford Network, have emerged as examples that offer collaborative frameworks for aggregating high-quality data to overcome issues of data scarcity and variability [12]. Challenges like clinical usability and model generalization can be addressed through explainable artificial intelligence (XAI), which enhances transparency and builds clinician trust [22]. This can be achieved by transparent data practices and clear disclosures about imbalanced datasets, which are essential for meeting the needs of underserved populations [90]. Additionally, integrating ethics review boards early in the AI development process can guide data governance and ensure compliance with privacy laws [12]. Addressing ethical challenges also requires diverse teams of researchers, engineers, informaticists, and neonatologists to tackle these problems using equity-focused frameworks, particularly for addressing historically understudied challenges in neonatal care. Multicenter validation and standardized data collection are also important to ensure model reliability and address variability across clinical environments [12]. Lastly, centralized AI platforms could be used to streamline model integration into workflows, support continuous updates, and address performance degradation over time [22]. Regular audits, along with clear documentation of model development and trade-offs, further ensure responsible implementation [90]. These strategies provide actionable pathways for advancing robust, ethical, and equitable AI adoption in NICUs, paving the way for impactful neonatal care.

Future directions for NICU opportunities present key research areas. First, integrating AI with medical imaging, especially for conditions like ROP, demands refined technology, larger datasets, and real-world validation. These challenges pertaining to data integration have also been highlighted in previous reviews [12,22]. Second, enhancing data-driven insights and predictive models requires broader clinical scenarios and improved data quality via collaborative NICU efforts. Building on recommendations in prior reviews to explore clinician trust in AI systems, this study highlights the need for actionable strategies to enhance transparency and multidisciplinary collaboration [21]. Understanding neonatal conditions better, exploring diverse risk factors, and fostering multidisciplinary collaborations are key priorities. Practical AI application in NICUs, optimizing resource allocation and care, requires real-world implementation and thorough assessment. Lastly, refining scalable personalized care plans and interventions, while ethically considering AI personalization, remains essential for expanding AI's role and improving neonatal outcomes.

### Conclusions

#### Opportunities and Challenges

This systematic review has highlighted substantial opportunities for AI in the NICU. Advancements in medical imaging, combined with AI, have the potential to improve diagnostic accuracy and enable early intervention in this field. Furthermore, data-driven insights and predictive models offer the opportunity to enhance clinical decision-making and deepen our understanding of neonatal conditions. Additionally, personalized neonatal care could optimize health care delivery for individual neonates.

Simultaneously, the study has revealed crucial challenges in integrating AI in the NICU. Issues related to data quality emphasize the need for robust data management strategies. Ensuring clinical interpretability and usability is essential to ensure AI tools align smoothly with clinical workflows, especially in the high-stress NICU environment. Moreover, achieving model generalization and validation across diverse patient populations and addressing clinical and diagnostic variability are essential considerations. Ethical and regulatory challenges, including data privacy, security, and model validation, underscore the importance of responsible AI adoption in the NICU.

These findings align with existing literature, revealing the unique complexities of the NICU context. The identified research gaps include the need for practical AI implementations within the NICU, considering resource constraints and clinical requirements. Additionally, there is a need for the development of ethical frameworks and regulatory guidelines tailored specifically to the NICU environment. Future research should focus on practical implementation, ethical frameworks, and regulatory guidelines to realize AI's potential in the NICU.

This research holds potential for neonatal care, benefiting a range of stakeholders. Health care professionals, including neonatologists, nurses, and other clinicians, can gain directly from the insights provided in this study. Health care institutions that manage NICUs also stand to benefit, as the research highlights the importance of practical AI implementation within the NICU, enabling them to optimize resource allocation and improve patient care. This research serves as an important resource for advancing AI technology in neonatal care, fostering a future of improved health care delivery and enhanced well-being for NICU patients and their families.

#### Limitations and Future Directions

The exploration of the potential of AI in the NICU to predict clinical outcomes and length of stay comes with a recognition of strengths and limitations that shape the scope and methodology of this study. The search strategy in this systematic review was designed to be comprehensive, incorporating a range of keywords and subject headings to capture relevant literature. The search terms were carefully chosen in collaboration with experienced research librarians to ensure inclusivity; however, the complexity of the AI field and evolving terminologies might have introduced limitations. It is possible that studies using emerging AI-related terminology were not captured. Furthermore, the inclusion criteria focused on academic research articles, which inadvertently excluded insights from private organizations and the grey literature. This limitation could result in missing valuable perspectives and data that could enhance understanding of AI applications in the NICU. Moreover, the decision to restrict the study to English full-text articles introduced a language bias, primarily focusing on studies from English-speaking countries. As a result, research conducted in other languages may have been excluded, limiting the diversity and global representation in this review. Additionally, this study's eligibility criteria specifically focused on interventions emphasizing predictive aspects of AI, which could have led to an incomplete portrayal of AI's multifaceted role in the NICU. Nonpredictive aspects of AI, while relevant, were not the central focus of this systematic review.

Despite these potential limitations, this study provides valuable insights into the challenges and opportunities in the emerging field of AI predictions in the NICU. The decision to focus on academic research articles ensured a rigorous examination of peer-reviewed literature, lending credibility to the findings. While recognizing these limitations, the study serves as a foundation for further exploration and analysis in this dynamic and evolving field. Our review considered studies published until March 2023, providing a snapshot of advancements in AI applications in NICUs during that period. Post the review period, new developments include the use of ensemble machine learning models for predicting NICU length of stay with high accuracy through hybrid approaches such as Classifier Fusion-LoS, Gradient Boosting, CatBoost, and Recurrent Neural Networks, which can result in better resource management and quality of care [91,92]. Additionally, researchers have developed advanced AI models, which can enable clinicians to interpret predictions for sepsis more effectively and accurately [93,94]. Furthermore, new research combining proteomics with explainable machine learning methods has facilitated the identification of biomarkers for critical conditions, such as posthemorrhagic ventricular dilation, offering enhanced early intervention capabilities in neonates [95]. While these advancements build upon the opportunities identified in our study, particularly in the domains of explainability, real-time applications, and personalized neonatal care, the studies also highlight the existing challenges including the lack of high-quality and comprehensive datasets and the need for clinician training to effectively use these AI tools [91-94].

The systematic review process has exposed several research gaps. First, there is a noticeable gap concerning the maturity level of AI implementation in NICUs. While existing studies have laid the foundation, further research is needed to extend AI maturity to the next stages of emerging and integration. Second, the scarcity of studies addressing ethical and regulatory aspects of AI in NICUs is evident. These areas, such as liability in cases of adverse outcomes resulting from AI predictions, warrant a comprehensive investigation. Furthermore, the absence of studies reporting unfavorable or null results suggests potential reporting bias within the field. Finally, collaborative partnerships and patient and family engagement are instrumental in advancing AI research in NICUs.

Overall, these future directions and improvements collectively contribute to the ongoing evolution of AI research in NICUs, fostering a more comprehensive understanding of the opportunities and challenges while refining the methodology for more effective and robust reviews in the future.

## Appendix

Multimedia Appendix 1 Full-text excluded studies—reasons.

Multimedia Appendix 2 Quality criteria checklist.

Multimedia Appendix 3 PRISMA 2020 checklist.

Multimedia Appendix 4 Included studies.

Multimedia Appendix 5 Clinical outcomes description.

## Abbreviations

AI: artificial intelligence
AUROC: area under the receiver operating characteristic curve
BPD: bronchopulmonary dysplasia
MeSH: Medical Subject Headings
MPSE: Mendelian Phenotype Search Engine
MRI: magnetic resonance imaging
NICU: neonatal intensive care unit
PH: pulmonary hypertension
PRISMA: Preferred Reporting Items for Systematic Reviews and Meta-Analyses
QCC: Quality Checklist for Clinical Case Series
RMSE: root mean square error
ROP: retinopathy of prematurity
WHO: World Health Organization
XAI: explainable artificial intelligence

## Data Availability

All data supporting the findings of this study are provided in the multimedia appendices included with this manuscript. Additional information or clarification can be requested from the corresponding author.
