# Supplementary material for: Opportunities and Challenges of Using Artificial Intelligence in Predicting Clinical Outcomes and Length of Stay in Neonatal Intensive Care Units: Systematic Review
Source: J Med Internet Res. 2025 Oct 3;27:e63175. doi: 10.2196/63175 (PMC12534773; doi:10.2196/63175)
Supplement: Multimedia Appendix 1 [file jmir_v27i1e63175_app1.docx]

# Full-Text Excluded Studies—Reasons

| **Title** | **Study** | **Reason** |
| --- | --- | --- |
| Evaluation of the clinical effect of an artificial intelligence-assisted diagnosis and treatment system for neonatal seizures in the real world: a multicenter clinical study protocol. | Xiao 2022 | Non-English |
| HeRO: AI with Evidence | King 2022 | Non-English |
| Alteration of skin condition in newborns admitted to neonatal intensive care: a concept analysis | Arajo 2022 | Non-English |
| Parasympathetic evaluation for procedural pain assessment in neonatology | BachillerCarnicero 2022 | Non-English |
| Computerized nursing process: development of a mobile technology for use with neonates | Lima 2018 | Non-English |
| Neonatal near miss in the intensive care unit | Maia 2020 | Non-English |
| Developmental Care: assistance of nurses from Neonatal Intensive Care Units | Marski 2018 | Non-English |
| Death and dying of newborns and children: relationships between nursing and family according to Travelbee | Medeiros 2022 | Non-English |
| Adherence of the nursing team to patient safety actions in neonatal units | Mendes 2021 | Non-English |
| Psychological stress in mothers of neonates admitted to an intensive care unit | Mesa 2021 | Non-English |
| Safety elements and understanding of guidelines at discharge from Neonatal Intensive Care Units | Pucca 2022 | Non-English |
| Assistance flowchart for pain management in a Neonatal Intensive Care Unit | Querido 2018 | Non-English |
| Incidence, predictors of success and outcome of LISA in very preterm infants | Balazs 2022 | Wrong intervention |
| Challenges in diagnosing necrotizing enterocolitis. | Kim 2020 | Wrong intervention |
| An agent based architecture for high-risk neonate management at neonatal intensive care unit. | Malak 2018 | Wrong intervention |
| A Call for Defining Pediatric Chronic Critical Illness: Moving beyond i Know It When i See It* | MurphySalem 2023 | Wrong intervention |
| Integrated Visualization Highlighting Retinal Changes in Retinopathy of Prematurity From 3-Dimensional Optical Coherence Tomography Data | Mangalesh 2022 | Wrong intervention |
| Respiratory Monitoring: Current State of the Art and Future Roads | Costanzo 2022 | Wrong intervention |
| Automated brain morphometric biomarkers from MRI at term predict motor development in very preterm infants | Kline 2020 | Wrong intervention |
| Predictive values of location and volumetric MRI injury patterns for neurodevelopmental outcomes in hypoxic-ischemic encephalopathy neonates | Chang 2020 | Wrong intervention |
| Perinatal mortality in Saudi Arabia: Profile from a private setup | Rahman 2020 | Wrong intervention |
| Erratum: Family Integrated Care (FICare) in Level II Neonatal Intensive Care Units: Study protocol for a cluster randomized controlled trial (Trials. (2017) 18 (467) DOI: 10.1186/s13063-017-2181-3) | Benzies 2020 | Wrong intervention |
| Optimizing timing of bilirubin screening the Neonatal Intensive Care Unit | Matsumoto 2019 | Wrong intervention |
| Predictive value of an early amplitude-integrated electroencephalogram for neurologic outcome in preterm infants | Park 2018 | Wrong intervention |
| Prospective study of early and late outcomes of extremely low birthweight in Central Saudi Arabia | Abolfotouh 2018 | Wrong intervention |
| Saudi Arabian retinopathy of prematurity national telemedicine program: Achievements and challenges | AlAmro 2022 | Wrong intervention |
| Prevalence, predictors, and outcomes of major congenital anomalies: A population-based register study | Al-Dewik 2023 | Wrong intervention |
| Characteristics of neonatal Sepsis at a tertiary care hospital in Saudi Arabia | Al-Matary 2019 | Wrong intervention |
| Traditional open bay neonatal intensive care units can be redesigned to better suit family centered care application | Al-Motlaq 2018 | Wrong intervention |
| Criteria for Using INSURE in Management of Premature Babies with Respiratory Distress Syndrome | Awaysheh 2019 | Wrong intervention |
| Neonatal Simulation Program: A 5 Years Educational Journey From Qatar | Bayoumi 2022 | Wrong intervention |
| Derivation and validation of a risk score to predict mortality of early neonates at neonatal intensive care unit: The END in NICU score | Belsti 2021 | Wrong intervention |
| Usability, acceptability, and feasibility of the Implementation of Infant Pain Practice Change (ImPaC) Resource | Bueno 2020 | Wrong intervention |
| Predicting the Severity and Outcome of Persistent Pulmonary Hypertension of the Newborn Using New Echocardiography Parameters | Butt 2022 | Wrong intervention |
| Necrotizing Enterocolitis and Its Predictors Among Preterm Neonates Admitted in Neonatal Intensive Care Units of Gurage Zone Public Hospitals, Southwest Ethiopia, 2021 | ChekoleTemere 2022 | Wrong intervention |
| Newborns‚Äô voice: We need pain management | Chen 2021 | Wrong intervention |
| Lung UltraSound Targeted Recruitment (LUSTR): A Novel Protocol to Optimize Open Lung Ventilation in Critically Ill Neonates | Chioma 2022 | Wrong intervention |
| Current status of laboratory and imaging diagnosis of neonatal necrotizing enterocolitis | D'Angelo 2018 | Wrong intervention |
| Pre-Vent: the prematurity-related ventilatory control study | Dennery 2019 | Wrong intervention |
| Vitamin A supplementation prevents the bronchopulmonary dysplasia in premature infants: A systematic review and meta-analysis | Ding 2021 | Wrong intervention |
| The potential effects of NICU environment and multisensory stimulation in prematurity | El-Metwally 2020 | Wrong intervention |
| Improving the Efficiency and Effectiveness of Parent Education in the Neonatal Intensive Care Unit | Gehl 2020 | Wrong intervention |
| Diagnostic, Prognostic, Predictive, and Monitoring Role of Neutrophil CD11b and Monocyte CD14 in Neonatal Sepsis | Hashem 2021 | Wrong intervention |
| An Update on the Prevention and Management of Bronchopulmonary Dysplasia | Hennelly 2021 | Wrong intervention |
| Near-Infrared spectroscopy for perfusion assessment and neonatal management | Hummler 2020 | Wrong intervention |
| Caregivers Help-Seeking Behaviors for Postdischarged Neonates from Neonatal Intensive Care Units: A Jordanian Study | Kasem 2020 | Wrong intervention |
| Cost-effectiveness analysis of heart rate characteristics monitoring to improve survival for very low birth weight infants | King 2022 | Wrong intervention |
| Multivariable Predictive Models of Death or Neurodevelopmental Impairment Among Extremely Low Birth Weight Infants Using Heart Rate Characteristics | King 2022 | Wrong intervention |
| Predictors of postoperative outcomes in infants with low birth weight undergoing congenital heart surgery: A retrospective observational study | Lu 2019 | Wrong intervention |
| Management of Extremity Venous Thrombosis in Neonates and Infants: An Experience From a Resource Challenged Setting | Mousa 2019 | Wrong intervention |
| A Novel Simulation Based Method For Training Pediatric & Neonatal Critical Care Transport Teams | Murdoch 2019 | Wrong intervention |
| Families‚Äô perspectives on monitoring infants‚Äô health and development after discharge from NICUs | O‚ÄôShea 2021 | Wrong intervention |
| Predictive value of an early amplitude-integrated electroencephalogram for short-term neurologic outcomes in preterm infants | Park 2020 | Wrong intervention |
| Newborn antibiotic exposures and association with proven bloodstream infection | Schulman 2019 | Wrong intervention |
| Causes and factors associated with neonatal mortality in Neonatal Intensive Care Unit (NICU) of Jimma University Medical Center, Jimma, South West Ethiopia | Seid 2019 | Wrong intervention |
| A Systematic Review and Pooled Prevalence of Paediatric Delirium in Critically Ill Children | Semple 2021 | Wrong intervention |
| Outcome prediction in newborn infants: Past, present, and future | Shukla 2022 | Wrong intervention |
| Perception of health professionals about neonatal palliative care | Silva 2019 | Wrong intervention |
| Formal procedure to facilitate the decision to withhold or withdraw life-sustaining interventions in a neonatal intensive care unit: A seven-year retrospective study | Sorin 2018 | Wrong intervention |
| Vancomycin is commonly under‚Äêdosed in critically ill children and neonates | Sosnin 2019 | Wrong intervention |
| Neonatal Intensive Care Unit Length of Stay Reduction by Heart Rate Characteristics Monitoring | Swanson 2018 | Wrong intervention |
| Regarding "Burden of Comorbidities and Healthcare Resource Utilization Among Medicaid-Enrolled Extremely Premature Infants" | Tsapatsaris 2023 | Wrong intervention |
| Impact of low‚Äêdose aspirin on adverse perinatal outcome: meta‚Äêanalysis and meta‚Äêregression | Turner 2020 | Wrong intervention |
| Optimisation of clinical workflow and monitor settings safely reduces alarms in the NICU | Varisco 2021 | Wrong intervention |
| Longitudinal microbiome composition and stability correlate with increased weight and length of very-low-birth-weight infants | Yee 2019 | Wrong intervention |
| The Ideal Timing of Bedside Surgical Ligation of Patent Ductus Arteriosus in Premature Babies Less Than 30 Gestational Weeks | Z√ºbarioƒülu 2021 | Wrong intervention |
| Machine Learning-Based Automatic Classification of Video Recorded Neonatal Manipulations and Associated Physiological Parameters: A Feasibility Study. | Singh 2020 | Wrong outcomes |
| Distinct effects of prematurity on MRI metrics of brain functional connectivity, activity, and structure: Univariate and multivariate analyses. | Chiarelli 2021 | Wrong outcomes |
| Identification of variation in nutritional practice in neonatal units in England and association with clinical outcomes using agnostic machine learning. | Greenbury 2021 | Wrong outcomes |
| Integration of an interpretable machine learning algorithm to identify early life risk factors of childhood obesity among preterm infants: a prospective birth cohort. | Fu 2020 | Wrong outcomes |
| Pilot Testing a Robot for Reducing Pain in Hospitalized Preterm Infants. | Williams 2019 | Wrong outcomes |
| Quiet sleep detection in preterm infants using deep convolutional neural networks. | Ansari 2018 | Wrong outcomes |
| Machine Learning-Based Automatic Classification of Video Recorded Neonatal Manipulations and Associated Physiological Parameters: A Feasibility Study. | Singh 2021 | Wrong outcomes |
| Researchers Use Health Data Tools to Rapidly Detect Sepsis in Sick Newborns. | Goldstein 2019 | Wrong outcomes |
| Predictive factors of the interruption of exclusive breastfeeding in premature infants: a prospective cohort | Luz 2018 | Wrong outcomes |
| Language function following preterm birth: prediction using machine learning. | Valavani 2022 | Wrong patient population |
| End-to-End Automatic Morphological Classification of Intracranial Pressure Pulse Waveforms Using Deep Learning. | Mataczynski 2022 | Wrong patient population |
| A machine-learning algorithm for neonatal seizure recognition: a multicentre, randomised, controlled trial. | Pavel 2020 | Wrong patient population |
| Feed-forward neural networks using cerebral MR spectroscopy and DTI might predict neurodevelopmental outcome in preterm neonates. | Janjic 2020 | Wrong patient population |
| Neonatal Brain Microstructure and Machine-Learning-Based Prediction of Early Language Development in Children Born Very Preterm. | Vassar 2020 | Wrong patient population |
| Point-of-Care MRI with Artificial Intelligence to Measure Midline Shift in Acute Stroke Follow-Up | Kundu 2022 | Wrong patient population |
| Continuous ECG monitoring should be the heart of bedside AI-based predictive analytics monitoring for early detection of clinical deterioration | Monfredi 2023 | Wrong patient population |
| Artificial intelligence and placental DNA methylation: newborn prediction and molecular mechanisms of autism in preterm children | Bahado-Singh 2022 | Wrong patient population |
| A personalized risk stratification tool for perinatal morbidity and mortality using explainable artificial intelligence (AI) | Zimmerman 2023 | Wrong patient population |
| The newborn delivery room of tomorrow: emerging and future technologies | Niemuth 2022 | Wrong patient population |
| Use of Artificial Intelligence in Obstetrics: not quite ready for prime time | Sarno 2022 | Wrong patient population |
| The Pursuit of Generalizability and Equity Through Artificial Intelligence-Based Risk Prediction Models | Oke 2022 | Wrong patient population |
| Assessment of germinal matrix hemorrhage on head ultrasound with deep learning algorithms | Kim 2022 | Wrong patient population |
| Newborn Eye Screening as an Application of AI | Kumm 2021 | Wrong patient population |
| Paradigm shift in medical education: The future beckons | Naithani 2021 | Wrong patient population |
| Ensemble machine learning accurately predicts pediatric ICU admission rates | Pelletier 2021 | Wrong patient population |
| 304 Predicting severe adverse perinatal outcomes in pregnancies complicated by fetal growth restriction: a validation study | Powel 2021 | Wrong patient population |
| The Deterioration Risk Index: Predicting Pediatric Inpatient Deterioration with Machine Learning and the Electronic Health Record | Gorham 2020 | Wrong patient population |
| Use of artificial intelligence (AI) in the interpretation of intrapartum fetal heart rate (FHR) tracings: a systematic review and meta-analysis | Balayla 2019 | Wrong patient population |
| Machine learning can accurately predict pre-admission baseline hemoglobin and creatinine in intensive care patients, bringing context to abnormal admission lab values | Dauvin 2019 | Wrong patient population |
| 407: Risk assessment for adverse neonatal outcomes in pregnancies complicated by gestational diabetes using machine learning | HOURI 2019 | Wrong patient population |
| Nursing factors associated with length of stay and readmission rate of the elderly residents from nursing home based on LTCfocus database. | Zhang 2022 | Wrong patient population |
| A natural language processing pipeline to advance the use of Twitter data for digital epidemiology of adverse pregnancy outcomes | Klein 2020 | Wrong patient population |
| Anxiety and depression in mothers of newborns in intensive care units | Souza 2021 | Wrong patient population |
| Viewpoint-Consistent 3D Face Alignment | Tulyakov 2018 | Wrong patient population |
| Overview of the neonatal research network: History, contributions, challenges, and future | Watterberg 2022 | Wrong study design |
| Artificial and human intelligence for early identification of neonatal sepsis. | Sullivan 2023 | Wrong study design |
| Biomarkers of necrotizing enterocolitis in the era of machine learning and omics. | Leiva 2023 | Wrong study design |
| Machine Learning Algorithms to Predict Mortality of Neonates on Mechanical Intubation for Respiratory Failure. | Hsu 2021 | Wrong study design |
| Machine Learning Approaches to Predict In-Hospital Mortality among Neonates with Clinically Suspected Sepsis in the Neonatal Intensive Care Unit. | Hsu 2021 | Wrong study design |
| Predicting clinical outcomes using artificial intelligence and machine learning in neonatal intensive care units: a systematic review. | McAdams 2022 | Wrong study design |
| Multilayer dynamic ensemble model for intensive care unit mortality prediction of neonate patients. | Juraev 2022 | Wrong study design |
| Developing a machine learning-based tool to extend the usability of the NICHD BPD Outcome Estimator to the Asian population. | Patel 2022 | Wrong study design |
| External Validation of a Retinopathy of Prematurity Screening Model Using Artificial Intelligence in 3 Low- and Middle-Income Populations. | Coyner 2022 | Wrong study design |
| Machine Learning Prediction Models for Neurodevelopmental Outcome After Preterm Birth: A Scoping Review and New Machine Learning Evaluation Framework. | vanBoven 2022 | Wrong study design |
| Artificial Intelligence for Retinopathy of Prematurity: Validation of a Vascular Severity Scale against International Expert Diagnosis. | Campbell 2022 | Wrong study design |
| Federated Learning for Multicenter Collaboration in Ophthalmology: Improving Classification Performance in Retinopathy of Prematurity. | Lu 2022 | Wrong study design |
| Artificial Intelligence to Improve Health Outcomes in the NICU and PICU: A Systematic Review. | Adegboro 2022 | Wrong study design |
| Identifying clinical phenotypes in extremely low birth weight infants-an unsupervised machine learning approach. | Matsushita 2022 | Wrong study design |
| Forecasting the length-of-stay of pediatric patients in hospitals: a scoping review. | Medeiros 2021 | Wrong study design |
| Machine Learning for Detection of Correct Peripherally Inserted Central Catheter Tip Position from Radiology Reports in Infants. | Shah 2021 | Wrong study design |
| Evaluation of a Deep Learning-Derived Quantitative Retinopathy of Prematurity Severity Scale. | Campbell 2021 | Wrong study design |
| Early Detection of Late Onset Sepsis in Premature Infants Using Visibility Graph Analysis of Heart Rate Variability. | Leon 2021 | Wrong study design |
| Convolutional Neural Network Based on Fluorescein Angiography Images for Retinopathy of Prematurity Management. | Lepore 2020 | Wrong study design |
| Development and use of an adjusted nurse staffing metric in the neonatal intensive care unit. | Tawfik 2020 | Wrong study design |
| Automated Fundus Image Quality Assessment in Retinopathy of Prematurity Using Deep Convolutional Neural Networks. | Coyner 2019 | Wrong study design |
| Estimation of Neonatal Intestinal Perforation Associated with Necrotizing Enterocolitis by Machine Learning Reveals New Key Factors. | Irles 2018 | Wrong study design |
| Automated Diagnosis of Plus Disease in Retinopathy of Prematurity Using Deep Convolutional Neural Networks. | Brown 2018 | Wrong study design |
| Using machine learning to impact on long-term clinical care: principles, challenges, and practicalities | Ashton 2023 | Wrong study design |
| Multiomics, artificial intelligence, and precision medicine in perinatology | Pammi 2023 | Wrong study design |
| A Continuous Late-Onset Sepsis Prediction Algorithm for Preterm Infants Using Multi-Channel Physiological Signals From a Patient Monitor | Peng 2023 | Wrong study design |
| Data analytics in a clinical setting: Applications to understanding breathing patterns and their relevance to neonatal disease | Wilson 2022 | Wrong study design |
| Clinical decision support in the neonatal ICU | Rao 2022 | Wrong study design |
| DeepLOS: Deep learning for late-onset sepsis prediction in preterm infants using heart rate variability | Peng 2022 | Wrong study design |
| Application of Machine Learning Approaches to Predict Postnatal Growth Failure in Very Low Birth Weight Infants | Han 2022 | Wrong study design |
| The principles of whole-hospital predictive analytics monitoring for clinical medicine originated in the neonatal ICU | RandallMoorman 2022 | Wrong study design |
| Comparison of Multivariable Logistic Regression and Machine Learning Models for Predicting Bronchopulmonary Dysplasia or Death in Very Preterm Infants | Khurshid 2021 | Wrong study design |
| Toward personalized medicine for pharmacological interventions in neonates using vital signs | Hartley 2021 | Wrong study design |
| Neonatal sepsis prediction through clinical decision support algorithms: A systematic review | Persad 2021 | Wrong study design |
| AI in the Intensive Care Unit: Up-to-Date Review | Nguyen 2021 | Wrong study design |
| Applications of advanced signal processing and machine learning in the neonatal hypoxic-ischemic electroencephalogram | Abbasi 2020 | Wrong study design |
| Machine learning in critical care: State-of-the-art and a sepsis case study | Vellido 2018 | Wrong study design |
| Hybridized neural networks for non-invasive and continuous mortality risk assessment in neonates | Baker 2021 | Wrong study design |
| Cardio-respiratory signal extraction from video camera data for continuous non-contact vital sign monitoring using deep learning | Chaichulee 2019 | Wrong study design |
| Neonatal intensive care decision support systems using artificial intelligence techniques: a systematic review | Malak 2019 | Wrong study design |
| Machine learning models for early sepsis recognition in the neonatal intensive care unit using readily available electronic health record data | Masino 2019 | Wrong study design |
| Prediction of mortality of premature neonates using neural network and logistic regression | Rezaeian 2022 | Wrong study design |
| Neonatal Bowel Sound Detection Using Convolutional Neural Network and Laplace Hidden Semi-Markov Model | Sitaula 2022 | Wrong study design |
| Current Status and Future Directions of Neuromonitoring With Emerging Technologies in Neonatal Care | Variane 2022 | Wrong study design |
| A Comprehensive and Context-Sensitive Neonatal Pain Assessment Using Computer Vision | Zamzmi 2022 | Wrong study design |
