# Supplementary material for: Opportunities and Challenges of Using Artificial Intelligence in Predicting Clinical Outcomes and Length of Stay in Neonatal Intensive Care Units: Systematic Review
Source: J Med Internet Res. 2025 Oct 3;27:e63175. doi: 10.2196/63175 (PMC12534773; doi:10.2196/63175)
Supplement: Multimedia Appendix 2 [file jmir_v27i1e63175_app2.docx]

# Quality Criteria Checklist

| Study | Was the research question clearly stated? | Was the selection of study subjects/patients free from bias? | Were study groups comparable? | Was method of handling withdrawals described? | Was blinding used to prevent introduction of bias? | Was the intervention, comparison, and any intervening factors described in detail? | Were outcomes clearly defined and the measurements valid and reliable? | Was the statistical analysis appropriate for the study design and type of outcome indicators? | Do the conclusions consider potential biases and limitations in the results? | Is bias due to study’s funding or sponsorship unlikely? | Quality rating |
| --- | --- | --- | --- | --- | --- | --- | --- | --- | --- | --- | --- |
| Iyer et al (2022) [30] | Yes | Yes | Yes | Unclear | Unclear | Yes | Yes | Yes | Yes | Yes | High |
| He et al (2023) [36] | Yes | Yes | Yes | Yes | Unclear | Yes | Yes | Yes | Yes | Yes | High |
| Ali et al (2022) [38] | Yes | No | Yes | Unclear | Yes | Yes | Yes | Yes | Yes | Yes | Medium |
| Lin et al (2022) [46] | Yes | Yes | Yes | Yes | Yes | Unclear | Yes | Unclear | Yes | Yes | Medium |
| Lee et al (2021) [45] | Yes | Yes | Yes | Unclear | Unclear | Yes | Yes | Yes | Yes | Yes | High |
| Chen et al (2021) [39] | Yes | Unclear | Yes | Unclear | Unclear | Yes | Yes | Yes | Yes | Yes | Medium |
| Gschwandtner et al (2020) [41] | Yes | Unclear | Yes | Yes | Yes | Yes | Yes | Unclear | Yes | Yes | Medium |
| He et al (2020) [43] | Yes | Yes | Yes | Yes | Yes | Yes | Yes | Unclear | Yes | Yes | High |
| Braun et al (2020) [28] | Yes | Yes | Yes | Yes | Yes | Yes | Yes | Yes | Yes | Yes | High |
| Choi et al (2020) [40] | Yes | Yes | Unclear | No | Unclear | Yes | Yes | Unclear | Yes | Yes | Medium |
| Saha et al (2020) [50] | Yes | Unclear | Unclear | Unclear | Unclear | Yes | Yes | Yes | Yes | Yes | Medium |
| Huang et al (2020) [44] | Yes | Unclear | Yes | Unclear | Unclear | Yes | Yes | Yes | Yes | Yes | Medium |
| Hamilton et al (2020) [29] | Yes | Unclear | Yes | Unclear | No | Yes | Yes | Yes | Yes | Yes | Medium |
| He et al (2018) [42] | Yes | Unclear | Yes | Unclear | Yes | Yes | Yes | Yes | Yes | Yes | Medium |
| Kausch et al (2022) [37] | Yes | Yes | Yes | Unclear | Unclear | Yes | Yes | Yes | Yes | Yes | High |
| Verder et al (2021) [34] | Yes | Unclear | Unclear | No | No | Yes | Yes | Unclear | Yes | Yes | Medium |
| Patel et al (2022) [48] | Yes | Yes | Yes | Unclear | Yes | Yes | Yes | Yes | Yes | Yes | High |
| Ruixiang et al (2021) [49] | Yes | Unclear | Unclear | Yes | Unclear | Unclear | Yes | Unclear | Unclear | Yes | Medium |
| Shalish et al (2017) [33] | Yes | Unclear | Yes | No | Unclear | Yes | Yes | Unclear | Yes | Yes | Medium |
| Sheikhtaheri et al (2021) [51] | Yes | Yes | Unclear | Yes | Yes | Unclear | Yes | Yes | Yes | Yes | Medium |
| Ofman et al (2019) [32] | Yes | Yes | Yes | Yes | Unclear | Yes | Yes | Yes | Yes | Yes | High |
| Amodeo et al (2021) [35] | Yes | Yes | Yes | Unclear | Unclear | Yes | Yes | Unclear | Yes | Yes | High |
| Kovacs et al (2021) [31] | Yes | No | Yes | Unclear | Unclear | Yes | Yes | Yes | Yes | Yes | Medium |
| Lure et al (2021) [47] | Yes | No | Yes | No | Yes | Unclear | Yes | Yes | Yes | Yes | Medium |
