# Supplementary material for: Opportunities and Challenges of Using Artificial Intelligence in Predicting Clinical Outcomes and Length of Stay in Neonatal Intensive Care Units: Systematic Review
Source: J Med Internet Res. 2025 Oct 3;27:e63175. doi: 10.2196/63175 (PMC12534773; doi:10.2196/63175)
Supplement: Multimedia Appendix 3 [file jmir_v27i1e63175_app3.docx]

| **Section and Topic** | **Item #** | **Checklist item** | **Description/Location** |
| --- | --- | --- | --- |
| **TITLE** | | |  |
| Title | 1 | Identify the report as a systematic review. | Identified (mentioned in title) |
| **ABSTRACT** | | |  |
| Abstract | 2 | See the PRISMA 2020 for Abstracts checklist. | Structured abstract written as per PRISMA guidelines at the start of the paper |
| **INTRODUCTION** | | |  |
| Rationale | 3 | Describe the rationale for the review in the context of existing knowledge. | The introduction section, specifically paragraph 7 provides detailed rationale of the rapid review, especially in the context of existing reviews. |
| Objectives | 4 | Provide an explicit statement of the objective(s) or question(s) the review addresses. | Research objective and question provided at the end of introduction section. |
| **METHODS** | | |  |
| Eligibility criteria | 5 | Specify the inclusion and exclusion criteria for the review and how studies were grouped for the syntheses. | Specified in the ‘eligibility criteria’ sub-section of the methods section. |
| Information sources | 6 | Specify all databases, registers, websites, organisations, reference lists and other sources searched or consulted to identify studies. Specify the date when each source was last searched or consulted. | Specified in Appendix 1 table 1. Databases (Embase, Medline, CINAHL, etc.) and search dates (Jan 2017–Mar 2023) are also included. |
| Search strategy | 7 | Present the full search strategies for all databases, registers and websites, including any filters and limits used. | Specified in Appendix 1 table 1. |
| Selection process | 8 | Specify the methods used to decide whether a study met the inclusion criteria of the review, including how many reviewers screened each record and each report retrieved, whether they worked independently, and if applicable, details of automation tools used in the process. | Methods/Results: Study selection described, including title/abstract screening and use of Covidence software, See ‘quality appraisal’ and ‘study selection’ section. |
| Data collection process | 9 | Specify the methods used to collect data from reports, including how many reviewers collected data from each report, whether they worked independently, any processes for obtaining or confirming data from study investigators, and if applicable, details of automation tools used in the process. | Methods/Results: Described in ‘quality appraisal’ and ‘study selection’ section. ‘Study selection describes use of excel template. |
| Data items | 10a | List and define all outcomes for which data were sought. Specify whether all results that were compatible with each outcome domain in each study were sought (e.g. for all measures, time points, analyses), and if not, the methods used to decide which results to collect. | Results: Study characteristics section, especially figure 2 provides list of outcomes e.g. technology intervention, health condition, , and maturity level. |
|  | 10b | List and define all other variables for which data were sought (e.g. participant and intervention characteristics, funding sources). Describe any assumptions made about any missing or unclear information. | Results: Study characteristics section, especially figure 2 provides list of other variables (design, participants, location). |
| Study risk of bias assessment | 11 | Specify the methods used to assess risk of bias in the included studies, including details of the tool(s) used, how many reviewers assessed each study and whether they worked independently, and if applicable, details of automation tools used in the process. | Methods: Modified WHO and Quality Criteria Checklist (QCC) used for quality assessment (QCC provided in appendix). |
| Effect measures | 12 | Specify for each outcome the effect measure(s) (e.g. risk ratio, mean difference) used in the synthesis or presentation of results. | Not applicable (qualitative synthesis). |
| Synthesis methods | 13a | Describe the processes used to decide which studies were eligible for each synthesis (e.g. tabulating the study intervention characteristics and comparing against the planned groups for each synthesis (item #5)). | Methods: Thematic synthesis methods described thematically by technology, outcome, opportunities, and challenges as described in the data extraction method. |
|  | 13b | Describe any methods required to prepare the data for presentation or synthesis, such as handling of missing summary statistics, or data conversions. | Not required especially as the review involves qualitative synthesis. |
|  | 13c | Describe any methods used to tabulate or visually display results of individual studies and syntheses. | Data presented in tables and through bar graphs/figures in the results section. |
|  | 13d | Describe any methods used to synthesize results and provide a rationale for the choice(s). If meta-analysis was performed, describe the model(s), method(s) to identify the presence and extent of statistical heterogeneity, and software package(s) used. | Methods: See data extraction, thematic synthesis using Thomas and Harden’s method. |
|  | 13e | Describe any methods used to explore possible causes of heterogeneity among study results (e.g. subgroup analysis, meta-regression). | Not applicable (qualitative synthesis). |
|  | 13f | Describe any sensitivity analyses conducted to assess robustness of the synthesized results. | Not applicable (qualitative synthesis). |
| Reporting bias assessment | 14 | Describe any methods used to assess risk of bias due to missing results in a synthesis (arising from reporting biases). | Risk of bias due to missing results (reporting bias) was not formally assessed, as no meta-analysis was conducted and the number of studies per outcome was small. However, we acknowledge in the limitation section the possibility of publication bias, particularly due to the absence of studies reporting negative or null results. |
| Certainty assessment | 15 | Describe any methods used to assess certainty (or confidence) in the body of evidence for an outcome. | Quality appraisal of individual studies was conducted using modified WHO criteria and the Quality Criteria Checklist, and limitations related to evidence strength were discussed in the manuscript. A formal certainty assessment of the body of evidence (e.g., using GRADE) was not performed due to the heterogeneity of study designs and outcomes. |
| **RESULTS** | | |  |
| Study selection | 16a | Describe the results of the search and selection process, from the number of records identified in the search to the number of studies included in the review, ideally using a flow diagram. | Results: PRISMA diagram provided in figure 1. |
|  | 16b | Cite studies that might appear to meet the inclusion criteria, but which were excluded, and explain why they were excluded. | Refer to Multimedia Appendix 9. Full-Text Excluded Studies Reasons. |
| Study characteristics | 17 | Cite each included study and present its characteristics. | Study Characteristics table provided in the appendices. |
| Risk of bias in studies | 18 | Present assessments of risk of bias for each included study. | Risk of bias results reported using Quality Criteria Checklist in the appendix. |
| Results of individual studies | 19 | For all outcomes, present, for each study: (a) summary statistics for each group (where appropriate) and (b) an effect estimate and its precision (e.g. confidence/credible interval), ideally using structured tables or plots. | Results: Outcomes summarised in text, tables, and figures. Summary of study characteristics, clinical outcomes, opportunity and challenge’s themes and findings provided in the appendix. |
| Results of syntheses | 20a | For each synthesis, briefly summarise the characteristics and risk of bias among contributing studies. | Results: Thematic synthesis includes study-level characteristics and limitations. Quality Criteria Checklist in appendix also provides an overview of limitations/risk of bias. |
|  | 20b | Present results of all statistical syntheses conducted. If meta-analysis was done, present for each the summary estimate and its precision (e.g. confidence/credible interval) and measures of statistical heterogeneity. If comparing groups, describe the direction of the effect. | Not applicable (qualitative synthesis). |
|  | 20c | Present results of all investigations of possible causes of heterogeneity among study results. | Not applicable (qualitative synthesis). |
|  | 20d | Present results of all sensitivity analyses conducted to assess the robustness of the synthesized results. | Not applicable (qualitative synthesis). |
| Reporting biases | 21 | Present assessments of risk of bias due to missing results (arising from reporting biases) for each synthesis assessed. | Item 14, mentioned in limitations |
| Certainty of evidence | 22 | Present assessments of certainty (or confidence) in the body of evidence for each outcome assessed. | Item 15, appendix 2 |
| **DISCUSSION** | | |  |
| Discussion | 23a | Provide a general interpretation of the results in the context of other evidence. | Discussion: Results compared to existing literature (esp. para 2). |
|  | 23b | Discuss any limitations of the evidence included in the review. | Limitations section details limitations of the evidence used in the review. |
|  | 23c | Discuss any limitations of the review processes used. | Limitations section details limitations of the study with regards to the processes used. |
|  | 23d | Discuss implications of the results for practice, policy, and future research. | Conclusion: Provided mainly in conclusion section, and partly in the discussion section. |
| **OTHER INFORMATION** | | |  |
| Registration and protocol | 24a | Provide registration information for the review, including register name and registration number, or state that the review was not registered. | not registered. |
|  | 24b | Indicate where the review protocol can be accessed, or state that a protocol was not prepared. | NA |
|  | 24c | Describe and explain any amendments to information provided at registration or in the protocol. | NA |
| Support | 25 | Describe sources of financial or non-financial support for the review, and the role of the funders or sponsors in the review. | Acknowledgments section |
| Competing interests | 26 | Declare any competing interests of review authors. | None declared/Competing interest section |
| Availability of data, code and other materials | 27 | Report which of the following are publicly available and where they can be found: template data collection forms; data extracted from included studies; data used for all analyses; analytic code; any other materials used in the review. | Provided as supplementary material/in appendix. |

*From:*  Page MJ, McKenzie JE, Bossuyt PM, Boutron I, Hoffmann TC, Mulrow CD, et al. The PRISMA 2020 statement: an updated guideline for reporting systematic reviews. BMJ 2021;372:n71. doi: 10.1136/bmj.n71. This work is licensed under CC BY 4.0. To view a copy of this license, visit <https://creativecommons.org/licenses/by/4.0/>
