# Supplementary material for: Opportunities and Challenges of Using Artificial Intelligence in Predicting Clinical Outcomes and Length of Stay in Neonatal Intensive Care Units: Systematic Review
Source: J Med Internet Res. 2025 Oct 3;27:e63175. doi: 10.2196/63175 (PMC12534773; doi:10.2196/63175)
Supplement: Multimedia Appendix 4 [file jmir_v27i1e63175_app4.docx]

# Included Studies

| **Title** | **Published year** | **Journal** | **Study** | **Type of study** | **Country** | **Number of participants** | **Type of technology** | **Outcome category** |
| --- | --- | --- | --- | --- | --- | --- | --- | --- |
| Bedside tracking of functional autonomic age in preterm infants. | 2022 | Pediatric research | Iyer et al (2022) [30] | Prospective study | Austria | 67 | Machine Learning | Growth & Development |
| Risk factors and machine learning prediction models for bronchopulmonary dysplasia severity in the Chinese population. | 2022 | World journal of pediatrics : WJP | He et al (2023) [36] | Retrospective study | China | 471 | Machine & Deep Learning | Respiratory |
| A self-training deep neural network for early prediction of cognitive deficits in very preterm infants using brain functional connectome data. | 2022 | Pediatric radiology | Ali et al (2022) [38] | Retrospective study | USA | 343 | Deep Learning | Growth & Development |
| Predicting in-hospital length of stay for very-low-birth-weight preterm infants using machine learning techniques. | 2022 | Journal of the Formosan Medical Association = Taiwan yi zhi | Lin et al (2022) [46] | Retrospective study | Taiwan | 2940 | Deep Learning | Length of Stay |
| Predicting mortality risk for preterm infants using random forest. | 2021 | Scientific reports | Lee et al (2021) [45] | Retrospective study | USA | 275 | Deep Learning | Mortality |
| Deep Learning for the Diagnosis of Stage in Retinopathy of Prematurity: Accuracy and Generalizability across Populations and Cameras. | 2021 | Ophthalmology. Retina | Chen et al (2021) [39] | Retrospective study | USA, India, Napal | 1252 | Deep Learning | Ophthalmological |
| Deep learning for estimation of functional brain maturation from EEG of premature neonates. | 2020 | Annual International Conference of the IEEE Engineering in Medicine and Biology Society. IEEE Engineering in Medicine and Biology Society. Annual International Conference | Gschwandtner et al (2020) [41] | Retrospective study | Austria | 43 | Deep Learning | Growth & Development |
| A multi-task, multi-stage deep transfer learning model for early prediction of neurodevelopment in very preterm infants. | 2020 | Scientific reports | He et al (2020) [43] | Prospective study | USA | 1226 | Deep Learning | Growth & Development |
| Trends in Neonatal Intensive Care Unit Utilization in a Large Integrated Health Care System. | 2020 | JAMA network open | Braun et al (2020) [28] | Retrospective study | USA | 39220 | Machine Learning | Length of Stay |
| Variability in Plus Disease Identified Using a Deep Learning-Based Retinopathy of Prematurity Severity Scale. | 2020 | Ophthalmology. Retina | Choi et al (2020) [40] | Retrospective study | USA | 871 | Deep Learning | Ophthalmological |
| Predicting motor outcome in preterm infants from very early brain diffusion MRI using a deep learning convolutional neural network (CNN) model. | 2020 | NeuroImage | Saha et al (2020) [50] | Prospective study | USA | 77 | Deep Learning | Growth & Development |
| Prediction of visual outcomes by an artificial neural network following intravitreal injection and laser therapy for retinopathy of prematurity. | 2020 | The British journal of ophthalmology | Huang et al (2020) [44] | Retrospective study | Taiwan | 60 | Deep Learning | Ophthalmological |
| Estimating risk of severe neonatal morbidity in preterm births under 32 weeks of gestation. | 2020 | The journal of maternal-fetal & neonatal medicine : the official journal of the European Association of Perinatal Medicine, the Federation of Asia and Oceania Perinatal Societies, the International Society of Perinatal Obstetricians | Hamilton et al (2020) [29] | Multicenter prospective study | USA | 1039 | Machine Learning | Other |
| Early prediction of cognitive deficits in very preterm infants using functional connectome data in an artificial neural network framework. | 2018 | NeuroImage. Clinical | He et al (2018) [42] | Prospective study | USA | 912 | Deep Learning | Growth & Development |
| Cardiorespiratory signature of neonatal sepsis: development and validation of prediction models in 3 NICUs | 2023 | Pediatric Research | Kausch et al (2022) [37] | Retrospective study | USA | 2494 | Machine & Deep Learning | Other |
| Bronchopulmonary dysplasia predicted at birth by artificial intelligence | 2020 | Acta paediatrica (Oslo, Norway : 1992) | Verder et al (2021) [34] | Multicenter prospective study | Denmark | 61 | Machine Learning | Respiratory |
| The criticality Index-mortality: A dynamic machine learning prediction algorithm for mortality prediction in children cared for in an ICU | 2022 | Frontiers in Pediatrics | Patel et al (2022) [48] | Retrospective study | USA | 8399 | Deep Learning | Mortality |
| Early Physical Linear Growth of Small-for-Gestational-Age Infants Based on Computer Analysis Method | 2021 | Journal of Healthcare Engineering | Ruixiang et al (2021) [49] | Retrospective study | USA |  | Deep Learning | Growth & Development |
| Prediction of Extubation readiness in extremely preterm infants by the automated analysis of cardiorespiratory behavior: study protocol. | 2017 | BMC Pediatrics | Shalish et al (2017) [33] | Multicenter prospective study | USA | 170 | Machine Learning | Other |
| Prediction of neonatal deaths in NICUs: development and validation of machine learning models. | 2021 | BMC Medical Informatics & Decision Making | Sheikhtaheri et al (2021) [51] | Retrospective study | Iran | 1762 | Deep Learning | Mortality |
| The discovery BPD (D-BPD) program: study protocol of a prospective translational multicenter collaborative study to investigate determinants of chronic lung disease in very low birth weight infants. | 2019 | BMC Pediatrics | Ofman et al (2019) [32] | Prospective study | Argentina | 325 | Machine Learning | Respiratory |
| A maChine and deep Learning Approach to predict pulmoNary hyperteNsIon in newbornS with congenital diaphragmatic Hernia (CLANNISH): Protocol for a retrospective study | 2021 | PLoS One | Amodeo et al (2021) [35] | Retrospective study | Italy | 56 | Machine & Deep Learning | Respiratory |
| Developing practical clinical tools for predicting neonatal mortality at a neonatal intensive care unit in Tanzania | 2021 | BMC pediatrics | Kovacs et al (2021) [31] | Prospective study | Tanzania | 165 | Machine Learning | Mortality |
| Using machine learning analysis to assist in differentiating between necrotizing enterocolitis and spontaneous intestinal perforation: A novel predictive analytic tool | 2021 | J Pediatr Surg | Lure et al (2021) [47] | Retrospective study | USA | 40 | Deep Learning | Other |
