# Supplementary material for: Opportunities and Challenges of Using Artificial Intelligence in Predicting Clinical Outcomes and Length of Stay in Neonatal Intensive Care Units: Systematic Review
Source: J Med Internet Res. 2025 Oct 3;27:e63175. doi: 10.2196/63175 (PMC12534773; doi:10.2196/63175)
Supplement: Multimedia Appendix 5 [file jmir_v27i1e63175_app5.docx]

# Clinical Outcomes Description

| **Category** | **Outcome** | **Description** | **Study** |
| --- | --- | --- | --- |
| Growth & Development | Cognitive Development | The mental processes and abilities that involve the acquisition, processing, and utilization of information from the environment. It includes various aspects of thinking, reasoning, memory, attention, problem-solving, decision-making, language comprehension, and other higher-order mental activities [55]. | [38] |
|  |  |  | [41] |
|  |  |  | [42] |
|  |  |  | [43] |
|  | Growth | The physiological process of physical development and maturation in preterm infants. It involves the increase in size, weight, and overall development of various body structures and functions as the infants progress through their neonatal period [56]. | [30] |
|  |  |  | [49] |
|  | Motor Outcomes | Motor outcomes refer to the assessment and evaluation of an individual's motor skills and abilities, which encompass movements, coordination, muscle strength, and overall physical function [57]. | [50] |
| Length of Stay | Length of Stay | These studies focus on investigating and predicting the length of hospital stays for NICU patients. | [28] |
|  |  |  | [46] |
| Mortality | Mortality | Mortality studies focus on understanding and predicting the likelihood of death among extremely and very preterm infants. | [31] |
|  |  |  | [45] |
|  |  |  | [48] |
|  |  |  | [51] |
| Ophthalmological | Retinopathy of Prematurity (ROP) | A potentially sight-threatening eye condition that affects two thirds of premature infants with very low birth weight (<1250 g at birth). It is characterized by abnormal blood vessel growth in the retina, which can lead to scarring and retinal detachment, potentially causing vision impairment or blindness if left untreated [58]. | [39] |
|  |  |  | [40] |
|  |  |  | [44] |
| Other | Extubation Readiness | Extubation readiness refers to the state of a patient being prepared and deemed suitable for the removal of an endotracheal tube that has been providing mechanical ventilation. Medical professionals assess various physiological and clinical parameters to determine if an infant is ready for extubation, aiming to ensure a successful transition to spontaneous breathing and avoid potential complications [59]. | [33] |
|  | Multiple (Mortality, Periventricular Leukomalacia (PVL), Intraventricular Haemorrhage (IVH), BPD, Necrotizing Enterocolitis (NEC) | Intraventricular hemorrhage (IVH) is a medical condition characterized by bleeding that occurs within the brain's ventricular system, particularly in the ventricles, which are fluid-filled cavities in the brain. IVH is most commonly seen in premature infants, especially those born very prematurely, and is considered a serious medical complication in neonatal care [60].  Periventricular leukomalacia (PVL) is a neurological disorder that primarily affects premature infants, especially those born very prematurely. It is characterized by damage to the white matter of the brain, particularly in the periventricular region, which is the area surrounding the brain's fluid-filled cavities called ventricles [61]. | [29] |
|  | Necrotizing Enterocolitis (NEC)/Spontaneous Intestinal Perforation (SIP) | NEC and SIP are two distinct but serious gastrointestinal conditions that primarily affect premature infants. NEC involves the inflammation and potential death of intestinal tissue [62], while SIP refers to the spontaneous rupture or perforation of the intestinal wall [63]. Both conditions pose significant health risks to neonates and require prompt medical attention. | [47] |
|  | Sepsis | Neonatal sepsis is a serious medical condition characterized by a systemic infection that occurs within the first 28 days of life in newborn infants. It is caused by the presence of bacteria, viruses, fungi, or other pathogens in the bloodstream or other body tissues. Neonatal sepsis can lead to a range of complications and health issues, including organ dysfunction, shock, and even mortality if not promptly diagnosed and treated [64]. | [37] |
| Respiratory | BPD | A chronic lung disorder that primarily affects premature infants, especially those born very preterm. It is characterized by inflammation, injury, and abnormal growth of the developing lung tissue, particularly the alveoli (air sacs) and bronchioles (small airways) [65]. BPD often occurs as a result of mechanical ventilation and oxygen therapy used to support the immature lungs of premature infants. | [36] |
|  |  |  | [32] |
|  |  |  | [34] |
|  | Pulmonary Hypertension | The elevated blood pressure within the arteries of the lungs in newborns. This condition can occur in premature infants and those with certain medical conditions, where the blood vessels in the lungs become narrow and constricted, leading to increased pressure in the pulmonary arteries. PH can strain the right side of the heart as it works harder to pump blood through the lungs, potentially compromising oxygenation, and overall cardiac function [66]. | [35] |
